# Gastroprotective, Antioxidant, Anti-Inflammatory, and Toxicological Evaluation of Stem Bark Extracts of *Vitellaria paradoxa* and *Parkia biglobosa*

**DOI:** 10.3390/ph18081184

**Published:** 2025-08-11

**Authors:** Brice Dangnon, Durand Dah-Nouvlessounon, S. M. Ismaël Hoteyi, Haziz Sina, Justinian Andrei Tomescu, Kouassi Jean-Michel Akpo, Maxime Machioud Sangare-Oumar, Adolphe Adjanohoun, Olubukola Oluranti Babalola, Emanuel Vamanu, Lamine Baba-Moussa

**Affiliations:** 1 Laboratory of Biology and Molecular Typing in Microbiology, Department of Biochemistry and Cell Biology, Faculty of Sciences and Technology, University of Abomey-Calavi, Cotonou 05 BP 1604, Benin; brice.dangnon@uac.bj (B.D.); durand.dahnouvlessounon@uac.bj (D.D.-N.); smihot@yahoo.fr (S.M.I.H.); sina.haziz@gmail.com (H.S.); 2 Faculty of Biotechnology, University of Agronomic Science and Veterinary Medicine, 59 Marasti Blvd, 1 District, 011464 Bucharest, Romania; 3 Research Development Patents Department, Hofigal Export Import S.A., No. 2 Intrarea Serelor Street, District 4, 042124 Bucharest, Romania; andrei.tomescu@hofigal.eu; 4 Laboratory of Physiology and Experimental Pharmacology, Department of Animal Physiology, Faculty of Sciences and Technology, University of Abomey-Calavi, Cotonou 06 BP 2584, Benin; jeanmichelakpo3@gmail.com (K.J.-M.A.); sangoumarfr@yahoo.fr (M.M.S.-O.); 5 Institut National des Recherches Agricoles du Bénin, Cotonou 01 BP 284, Benin; adjanohouna@yahoo.fr; 6 Food Security and Safety Focus Area, Faculty of Natural and Agricultural Sciences, North-West University, Mmabatho 2735, South Africa; olubukola.babalola@nwu.ac.za

**Keywords:** phytochemicals, gastroprotection, oxidative stress, *Vitellaria paradoxa* extract, *Parkia biglobosa* extract

## Abstract

**Background/Objectives**: Oxidative stress is a pathophysiological factor that causes challenging issues in the treatment of several diseases, including gastric ulcer, inflammatory diseases, and adenocarcinomas. *V. paradoxa* and *P. biglobosa* are African plants whose parts are used for treating diseases, including gastrointestinal pathologies. This study aimed to characterize the gastroprotective, antioxidant, and anti-inflammatory activities of *V. paradoxa* and *P. biglobosa* stem bark extracts based on various solvents. **Methods**: The phytochemical screening and antioxidant evaluation were performed using radical scavenging (ABTS and DPPH) and reduction (FRAP and APM) methods. The anti-inflammatory activity was performed through an egg albumin denaturation model. The toxicological evaluation was performed on *Artemia salina* and female Wistar rat models, and the gastroprotective activity was carried out on an ethanolic-induced gastric ulcer rat model. **Results**: The results reported that *V. paradoxa* stem bark extracts contain catechin, epicatechin, ferulic acid, apigenin-7-gluc, and hesperidin, while *P. biglobosa* bark contains chlorogenic acid, catechin, caffeine, epicatechin, and cichoric acid. In the DPPH assay, the lowest scavenging capacities were 1.8 ± 0.21 mmol AAE/mg of dry extract (*V. paradoxa*, 97% ethanol) and 11.43 ± 0.208 mmol AAE/mg of dry extract (*P. biglobosa*, 50% ethanol). Similarly, for ABTS, the lowest scavenging capacities were 0.9726 ± 0.03952 mmol AAE/mg of dry extract (*V. paradoxa*, methanol with 1% HCl) and 1.3 mmol AAE/mg of dry extract (*P. biglobosa*, 97% ethanol), indicating strong antioxidant capacity. In the FRAP assay, both species reached a maximum reducing power of 2.39 mMol AAE/mg of dry extract (methanolic extract for *V. paradoxa*; methanol + 1% HCl for *P. biglobosa*). For APM, the 97% ethanolic extracts again showed the highest total antioxidant capacities: 31.78 ± 1.481 mMol AAE/mg (*V. paradoxa*) and 31.21 ± 0.852 mMol AAE/mg (*P. biglobosa*). The stem bark extracts of both *V. paradoxa* and *P. biglobosa* were revealed to be harmless in the *Artemia salina* as well as the rat model. The extracts of *V. paradoxa* as well as *P. biglobosa* exerted a stronger gastroprotective effect than omeprazole, a commonly used reference molecule. **Conclusions**: These extracts, rich in compounds exhibiting strong antioxidant, anti-inflammatory, and gastroprotective activities, surpassed omeprazole in ulcer protection in rat models. Their safety was confirmed in both *Artemia salina* and rodent assays. Future studies will explore their immunomodulatory, antiproliferative activities in vitro and in vivo and, specifically, the efficacy of isolated compounds in gastric adenocarcinoma models to assess these plants’ anticancer potential and elucidate their underlying mechanisms.

## 1. Introduction

The excessive production of reactive oxygen species (ROS) and reactive nitrogen species (RNS) that surpasses the body’s natural antioxidant defenses leads to oxidative stress, which has significant implications for biological systems [1,2,3]. As a result, oxidative stress occurs when there is an imbalance between the body’s oxidizing systems, mainly composed of free radicals, and the levels of ROS and RNS [4,5].

ROS are unstable molecules produced by all cells, including hydrogen peroxide (H_2_O_2_), hydroxyl radical (•OH), singlet oxygen (^1^O_2_), and superoxide (O^−2^). They are involved in deoxyribonucleotide formation, prostaglandin production, and reactions like oxidation, carboxylation, and hydroxylation, which are essential for cell function [6]. ROS also participate in defending the host against microbial infections, regulating vascular tone and cell adhesion, and acting as sensors for oxygen levels [7]. Moreover, they are involved in inflammation, not only as mediators but more importantly as regulators of cell signaling, promoting either cell proliferation and survival or cell death [8]. ROS play a key role as signaling molecules in the development of inflammation, which is considered an immunoregulator [9].

Recent findings emphasize the critical role of oxidative stress in initiating and maintaining inflammation, thereby affecting the development of many serious conditions, including cardiovascular diseases, diabetes, cancer, and neurodegenerative disorders [2,3,10,11,12,13]. The disruption of redox balance is linked to the development of various gastrointestinal disorders, such as Barrett’s esophagus, peptic ulcers, celiac disease, inflammatory bowel disease, and several types of adenocarcinomas [3,14]. Additionally, viral, bacterial, and parasitic infections are known to trigger ROS production, while also inducing and modulating antioxidant defense systems to create favorable environments for their spread [15,16]. For example, the gastrointestinal tract is a highly sensitive mucosal tissue exposed to external pathogens from food, which causes the stomach and intestines to produce oxidative stress; this response may be related to the emergence of gastrointestinal conditions including gastroenteritis, gastric ulcers, gastric and colon cancers, and functional disorders like functional dyspepsia [17]. In fact, gastrointestinal pathogens significantly contribute to oxidative stress and the breakdown of mucosal barriers [17,18]. Some viruses can manipulate the body’s redox system; for example, coronaviruses cause downregulation of antioxidant genes like Nuclear Factor erythroid 2-related factor 2 (NRF 2), while increasing the expression of oxidative stress-related genes such as myeloperoxidase, potentially promoting both viral replication and inflammation [19,20]. Oxidative stress is also closely linked to aging and overall mortality [10,21], with reports indicating a positive correlation between oxidative stress levels and long-term as well as all-cause mortality [21,22]. Higher oxidative stress levels are also associated with increased mortality risk among older women [23]. Despite the proven effectiveness of traditional treatments like proton pump inhibitors (PPIs), non-steroidal anti-inflammatory drugs (NSAIDs), corticosteroids, and synthetic antioxidants, their long-term use raises important clinical concerns. For example, PPIs have been linked to adverse effects, including hypomagnesemia, opportunistic infections, and poor nutrient absorption [24]. NSAIDs and corticosteroids are known to cause gastrointestinal ulcers, bleeding, immunosuppression, and other serious systemic issues [25,26]. Furthermore, certain synthetic antioxidants, such as butylated hydroxyanisole (BHA) and butylated hydroxytoluene (BHT), have shown potential toxicity and are suspected of causing birth defects [27].

These limitations highlight the urgent need for safer, more effective, and better-tolerated therapeutic options. In this context, medicinal plants have gained increasing attention due to their rich contents of natural bioactive compounds with proven anti-inflammatory, antioxidant, and gastroprotective properties [28,29].

Recent interest in plant-based remedies for ulcer treatment has underscored the potential of natural immunomodulators [30,31]. Additionally, the use of natural products in cancer therapy and as antioxidants has gained recognition due to their effectiveness [32,33,34]. Natural antioxidants have proven useful as medicinal supplements and alternative drugs that can prevent or protect the body against oxidative stress-related diseases, as well as food additives that inhibit lipid peroxidation [34]. Plants offer sustainable, safer, and more effective antioxidant options, mainly due to their phenolic compounds, which are a notable strategy for reducing pathological conditions [35,36]. Exploring these natural resources could thus provide a promising pathway for developing complementary or alternative treatments with enhanced safety profiles.

*P. biglobosa* (Jacq.) Benth., also known as African locust bean, is a common plant in the Western African region that has been traditionally used for various medicinal purposes, such as antidiabetic, anti-hypertensive, anti-inflammatory, wound healing, antihelmintic, and antifungal applications [37,38,39,40]. In Benin, it has multiple uses, including medicinal, handicraft, domestic, medico-magic, veterinary, cultural, food, and commercial applications, with the most-used parts being pulp, seeds, and bark [40,41]. It is reported to contain phytochemicals such as saponins, tannins, flavonoids, terpenoids, alkaloids, cardiac glycosides, and coumarins [40,42,43,44,45,46,47,48,49]. Additionally, it possesses antidiabetic, antimalarial [47], antioxidant [44], antibacterial [43,50], antifungal [51], antihypertensive, anti-inflammatory, anti-carcinogenic, anti-nociceptive, and analgesic activities [40,49,52]. Furthermore, *P. biglobosa* is claimed to inhibit DPPH, ABTS, and CUPRAC, and also to inhibit several enzymes (α-Glucosidase; α-amylase and cholinesterase) in vitro [42,48,53,54]. Moreover, Ajibola et al. [55] reported the hepatoprotective effect of *P. biglobosa* stem bark methanolic extract against paracetamol-induced liver damage in Wistar rats.

In parallel, *V. paradoxa*, also known as the shea tree, is a plant commonly found in West Africa. Reported to contain polyphenolic compounds [39,45,46,56,57,58], it is a plant traditionally used to treat diarrhea with blood, as a tonic and an appetizer, and to treat yellow fever, bee and wasp stings, waist pain, skin problems, rheumatism, bone dislocations and fractures, back pain, and arthritis, as well as being used as a pain-reliever [59,60]. Recent scientific studies have shown *V. paradoxa* to demonstrate anticancer [61,62,63], melanogenesis-inhibitory, antibacterial, antidiabetic, anti-inflammatory [64], anti-arthritic, anti-diarrheal, antimalarial, antigonorrheal (inhibition of Nesseria gonorrhoeae strains) [61], antireverse transcriptase [61], antioxidant activity (ABTS, DPPH, FRAP, CUPRAC, nitric oxide, and phosphomolybdenum assays) [46,62], enzyme inhibition (amylase, tyrosinase, and glucosidase), wound healing, intoxication prevention, antibacterial, antimycobacterial, and antifungal activities [39,56,57,63,64,65,66,67,68,69,70,71,72,73,74,75].

The aforementioned studies have assessed the various biological activities of *V. paradoxa* and *P. biglobosa* extracts. However, the integration of traditional medicinal plants such as *Vitellaria paradoxa* (shea tree) and *Parkia biglobosa* (African locust bean), along with phytochemical screenings, a determination of their antioxidant capacity, toxicological studies, and gastroprotective capacity evaluations, have not been demonstrated [76]. The combination of *Vitellaria paradoxa* and *Parkia biglobosa* extracts, along with the specific assays conducted in this study, represent a novel contribution to the literature. This combination and the parameters tested have not been previously reported.

The aim of this research was to characterize the toxicity, gastroprotective effects, and antioxidant and anti-inflammatory activities of *V. paradoxa* and *P. biglobosa* stem bark extracts using various solvents. We examined the phenolic contents, toxicity, in vitro antioxidant activity, and the in vitro anti-inflammatory and gastroprotective effects of the stem bark extracts from both plants.

## 2. Results

### 2.1. Phytochemical Screening of V. paradoxa and P. biglobosa Stem Bark

The results of the phytochemical screening of *V. paradoxa* and *P. biglobosa* stem bark powders are summarized in Table 1. These results show that *V. paradoxa* and *P. biglobosa* stem barks contain coumarins; tannins, including gallic tannins and catechic tannins; and flavonoids, such as anthocyanins and leuco-anthocyanins in the case of *V. paradoxa*, and anthocyanins in the case of *P. biglobosa*. They also contain saponosides, terpenic compounds including triterpenoids, and mucilages. Alkaloids were only detected in *V. paradoxa* stem bark powder, while glycosides such as O-heterosides with reduced genine and C-heterosides were only found in *P. biglobosa* stem bark powder.

### 2.2. Polyphenolic Content of Extracts from the Stem Bark of V. paradoxa and P. biglobosa

#### 2.2.1. Total Phenolic Content of *V. paradoxa* and *P. biglobosa* Stem Bark Extracts

**Figure 1:**
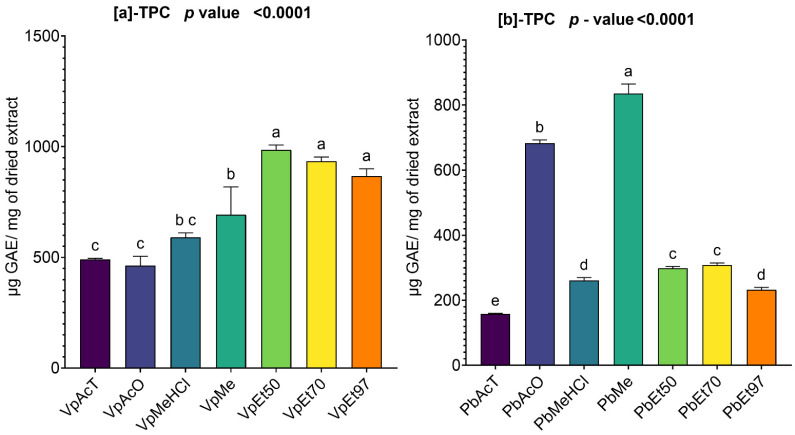
Total phenolic content (TPC) of stem bark of *V. paradoxa* (**a**) and *P. biglobosa* (**b**). Means with the same coefficients a, b, c, d and e are not statistically different.

The total phenolic contents of the bark extracts of *V. paradoxa* and *P. biglobosa* are summarized in Figure 1. The total phenolic content of the bark extracts of V. paradoxa varies depending on the extraction solvent (Figure 1a). The highest content, 986.46 ± 17.58 µg GAE/mg of dry extract, is observed in the extract obtained with 50% ethanol. This is followed by the extracts with 70% ethanol and 97% ethanol, which have respective contents of 933.7 ± 16.64 µg GAE/mg of dry extract and 867.67 ± 27.02 µg GAE/mg of dry extract. The extracts with ethyl acetate and acetone show a total phenolic content that is half of that found in the ethanol-based extracts. For the extracts based on methanol and methanol with 1% hydrochloric acid, total phenolic contents of 692.8 ± 102 µg GAE/mg of dry extract and 591.46 ± 15.97 µg GAE/mg of dry extract were recorded, respectively (Figure 1a).

The ANOVA analysis, conducted at a 95% significance level, indicated that the total phenolic contents of the ethanolic extracts are statistically similar. Additionally, at the same significance level, there was no significant statistical difference between the total phenolic contents of the extracts based on methanol and methanol with 1% hydrochloric acid. The same applies to extracts based on ethyl acetate, acetone, and methanol with 1% hydrochloric acid. However, with a *p*-value < 0.0001, the other differences observed are statistically significant (Figure 1a).

Regarding the stem bark extracts of *P. biglobosa*, acetone and methanol are the two solvents that extract the highest amount of total phenolics, with respective values of 682.8 ± 8.22 µg GAE/mg of dry extract and 835.5 ± 24 µg GAE/mg of dry extract. The extract with ethyl acetate shows the lowest total phenolic content, at 158 ± 1.63 µg GAE/mg of dry extract. The ethanolic extracts and the one with methanol and 1% hydrochloric acid contain roughly a third of the phenolics found in the acetone- and methanol-based extracts (Figure 1b).

The ANOVA analysis conducted at a 95% confidence level reported that the total phenolic contents of the extracts based on 50% and 70% ethanol are not statistically significant. Additionally, there is no statistically significant difference between the total phenolic contents of the extract based on methanol with 1% HCl and all the ethanolic extracts. All other observed differences are statistically significant (*p* value < 0.0001) (Figure 1b).

#### 2.2.2. Total Flavonoid Content of *V. paradoxa* and *P. biglobosa* Stem Bark Extracts

**Figure 2:**
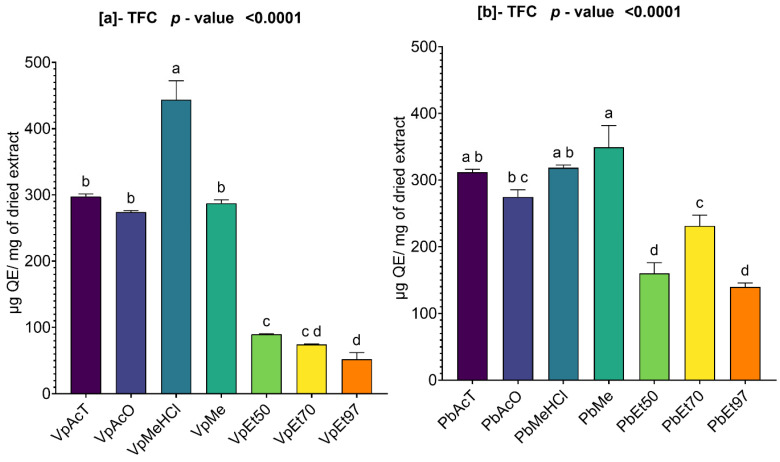
Total flavonoid content (TFC) in the stem bark of *V. paradoxa* (**a**) and *P. biglobosa* (**b**). Means sharing the same letters a, b, c and d are not significantly different.

The total flavonoid contents of the bark extracts of *V. paradoxa* and *P. biglobosa* are summarized in Figure 2. The figure shows how the total flavonoid contents vary depending on the extraction solvent.

For the bark extracts of *V. paradoxa*, the highest content (443.79 ± 23.42 µg QE/mg of dry extract) was observed in the extract based on methanol with 1% HCl, while the lowest content (52.08 ± 8.32 µg QE/mg of dry extract) was found in the extract based on 97% ethanol (Figure 2a). The ANOVA analysis performed at a 95% significance level revealed that there is no statistically significant difference among the total flavonoid contents of the ethanolic extracts, regardless of the percentage. Additionally, there was no statistically significant difference among the total flavonoid contents of the extracts based on ethyl acetate, acetone, and methanol. However, all other observed differences are statistically significant (*p* value < 0.0001) (Figure 2a).

Regarding the bark extracts of *P. biglobosa*, the methanol-based extract was the only one that concentrated the highest total flavonoid content, with a value of 349.37 ± 26.5 µg QE/mg of dry extract. The lowest total flavonoid content was 139.6 ± 4.93 µg QE/mg of dry extract (Figure 2b). An ANOVA analysis conducted at a 95% significance level did not show any statistically significant difference in total flavonoid contents among the extracts based on ethyl acetate, methanol, and methanol with 1% HCl. The same holds true for the extracts based on acetone and methanol with 1% HCl, ethyl acetate and acetone, acetone alone, and 50% and 97% ethanol. However, all other differences observed are statistically significant (*p* value < 0.0001) (Figure 2b).

#### 2.2.3. Total Condensed Tannins in *V. paradoxa* and *P. biglobosa* Stem Bark Extracts

**Figure 3:**
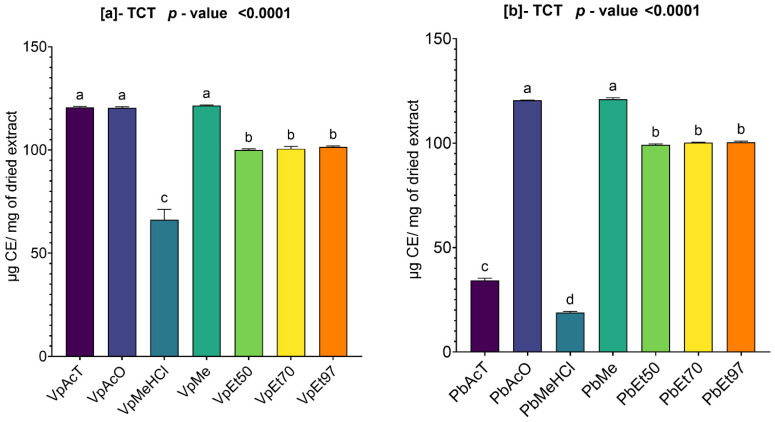
Total condensed tannins (TCT) in *V. paradoxa* (**a**) and *P. biglobosa* (**b**) stem bark extracts. Means sharing the same letters a, b, c or d are not statistically different.

The condensed tannin levels in the bark extracts of *V. paradoxa* and *P. biglobosa* are shown in Figure 3. A high content of condensed tannins was found in the extracts from both plant types.

The extracts based on ethyl acetate, acetone, and methanol showed the highest contents, with values of 120.682 µg CE/mg of dry extract, 120.437 µg CE/mg of dry extract, and 121.471 µg CE/mg of dry extract, respectively, for the bark of *V. paradoxa* (Figure 3a). The lowest condensed tannin content (66.104 µg CE/mg of dry extract) was observed in the extract based on methanol with 1% HCl (Figure 3a). The ANOVA test at a 95% significance level indicated that there is no statistically significant difference between the condensed tannin contents of the ethanolic extracts, regardless of the percentage, and between the extracts based on ethyl acetate, acetone, and methanol (Figure 3a). However, all other observed differences are statistically significant (*p* value < 0.0001) (Figure 3a).

For the extracts of *P. biglobosa*, acetone and methanol are the two solvents that yielded the highest condensed tannin contents, with respective values of 120.504 µg CE/mg of dry extract and 121.004 µg CE/mg of dry extract. Methanol with 1% HCl had the lowest condensed tannin content, with a value of 18.826 µg CE/mg of dry extract (Figure 3b). The ANOVA analysis performed at a 95% significance level indicated that, except for the statistical similarities found between the ethanolic extracts regardless of percentages and between the extracts based on acetone and methanol, all other observed differences are statistically significant (*p* value < 0.0001) (Figure 3b).

#### 2.2.4. Total Hydrolyzable Tannins in *V. paradoxa* and *P. biglobosa* Stem Bark Extracts

**Figure 4:**
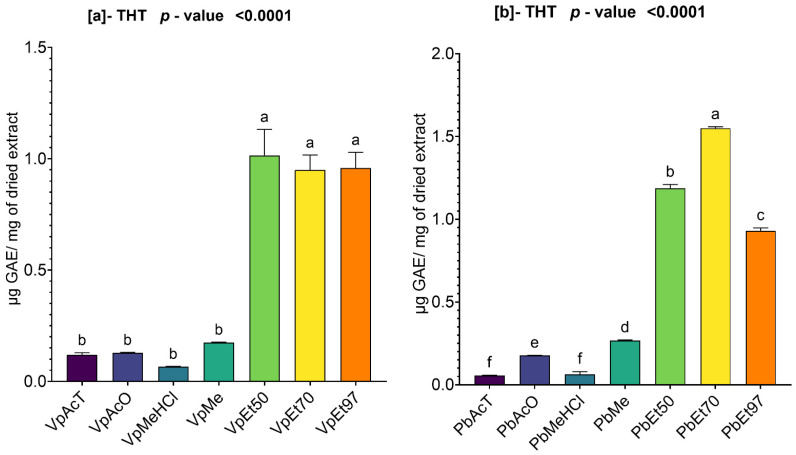
Total hydrolyzable tannins (THT) content of *V. paradoxa* (**a**) and *P. biglobosa* (**b**) stem bark extracts. Means sharing the same letters a, b, c, d, e or f are not statistically different.

The hydrolyzable tannin contents of the different bark extracts of *V. paradoxa* and *P. biglobosa* are shown in Figure 4. The analysis of this figure revealed that, for both *V. paradoxa* and *P. biglobosa*, the ethanolic extracts had the highest hydrolyzable tannin contents.

For the bark of *V. paradoxa*, 50% ethanol was the solvent with the highest hydrolyzable tannin content, measuring 1.01 ± 0.095 µg GAE/mg of dry extract. In contrast, the lowest content (0.06 ± 0.001 µg GAE/mg of dry extract) was found in the extract prepared with methanol containing 1% HCl (Figure 4a). The analysis of variance (ANOVA) conducted at a 95% significance level showed no statistically significant difference in hydrolyzable tannin levels among the ethanolic extracts, regardless of concentration. The same lack of significance applies to the extracts using ethyl acetate, acetone, methanol, and methanol with 1% HCl. However, the other differences observed are statistically significant (*p* value < 0.0001) (Figure 4a).

For the bark of *P. biglobosa*, the highest hydrolyzable tannin content, with a value of 1.54 ± 0.006 µg GAE/mg of dry extract, was recorded in the extract based on 70% ethanol, while the lowest content (0.05 ± 0.002 µg GAE/mg of dry extract) was found in the extract based on ethyl acetate (Figure 4b). The statistical analysis (ANOVA) conducted at a 95% significance level showed that, except for the extracts based on ethyl acetate and methanol with 1% HCl, which had statistically identical contents, all other differences observed were statistically significant (*p* < 0.0001) (Figure 4b).

#### 2.2.5. Polyphenolic Profiling and Quantification in the Stem Bark of *V. paradoxa* and *P. biglobosa* by HPLC-DAD

##### HPLC-DAD Determination of Phenolic Compounds in *V. paradoxa* Stem Bark

**Figure 5:**
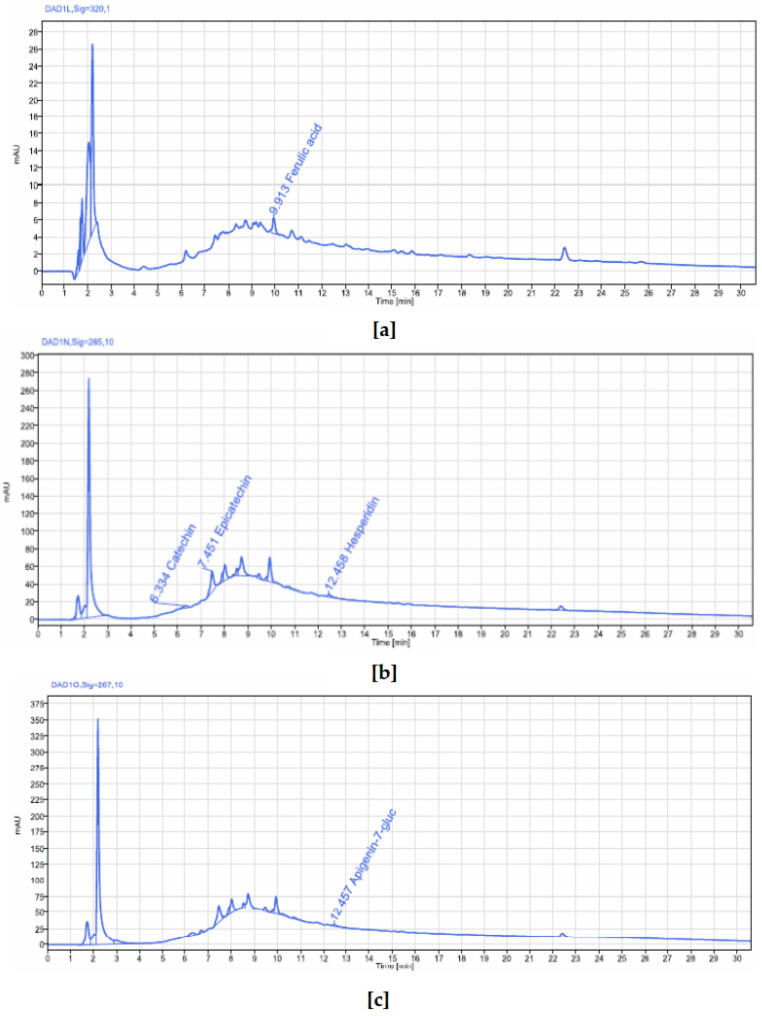
HPLC-DAD chromatograms of *V. paradoxa* stem bark extract: (**a**)—320 nm; (**b**)—285 nm; and (**c**)—267 nm.

An HPLC-DAD analysis of *V. paradoxa* stem bark extract was performed to identify phenolic compounds. The results are shown in the chromatograms in Figure 5. Five peaks were detected, corresponding to five different compounds, at wavelengths between 267 nm and 320 nm. These compounds include catechin, epicatechin, ferulic acid, apigenin-7-glucuronide, and hesperidin (Table 2). Among these, catechin, epicatechin, and apigenin-7-glucuronide had concentrations of 0.0641 µg/g, 0.3848 µg/g, and 0.012 µg/g of plant powder, respectively, while ferulic acid and hesperidin were below the quantification limits. It is also noteworthy that several other peaks, even those with the highest readings on the chromatograms at these wavelengths, were not identified or quantified (Figure 5).

##### HPLC-DAD Determination of Phenolic Compounds in *P. biglobosa* Stem Bark

**Figure 6:**
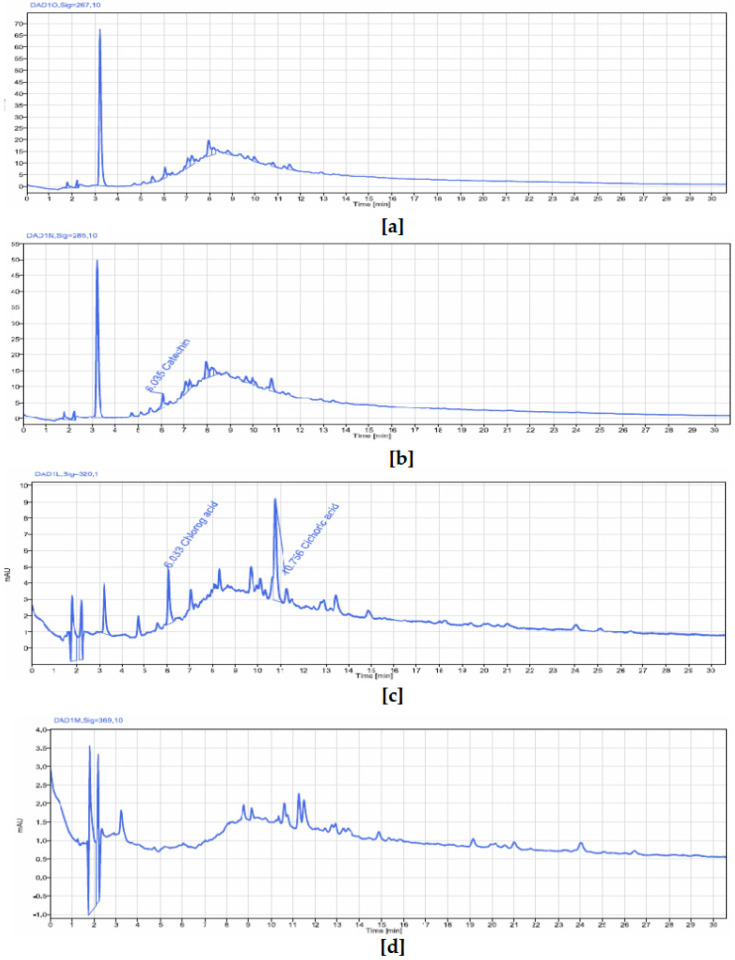
HPLC-DAD chromatogram of *P. biglobosa* stem bark extract: (**a**)—267 nm; (**b**)—285 nm; (**c**)—320 nm; (**d**)—369 nm.

**Figure 7:**
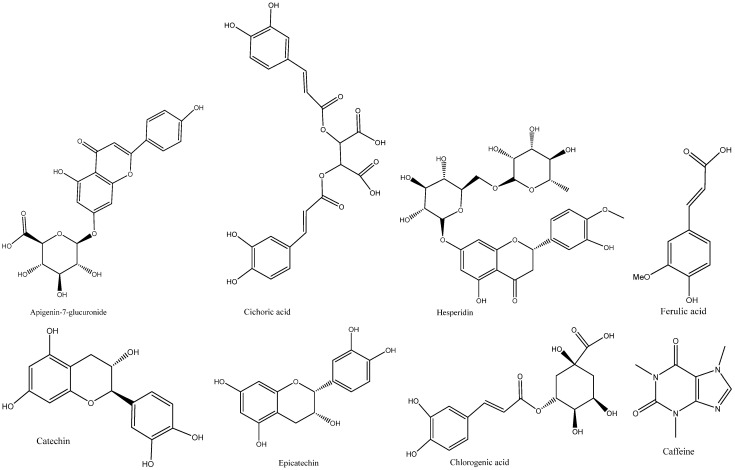
Molecular structure of phenolic compounds identified in the stem bark of *V. paradoxa* and *P. biglobosa*.

An HPLC-DAD analysis of *P. biglobosa* stem bark extract was conducted to identify phenolic compounds. Figure 6 and Table 3 display chromatograms summarizing the results of this analysis. Similarly to *V. paradoxa* stem bark extract, five peaks were detected and quantified at absorption signals between 267 and 320 nm. These peaks corresponded to compounds such as chlorogenic acid, catechin, caffeine, epicatechin, and cichoric acid (whose molecular structure is shown in Figure 7). Among these, chlorogenic acid, catechin, epicatechin, and cichoric acid had concentrations of 0.0055 µg/g, 0.0529 µg/g, 0.0423 µg/g, and 0.0166 µg/g of plant powder, respectively. The caffeine level was below the quantification limit. It is also worth noting that many peaks, including the highest, were not identified or quantified by the system.

### 2.3. Antioxidant Capacity of V. paradoxa and P. biglobosa Stem Bark Extract

#### 2.3.1. ABTS Scavenging Activity of Ethanolic Extract of *V. paradoxa* and *P. biglobosa*

**Figure 8:**
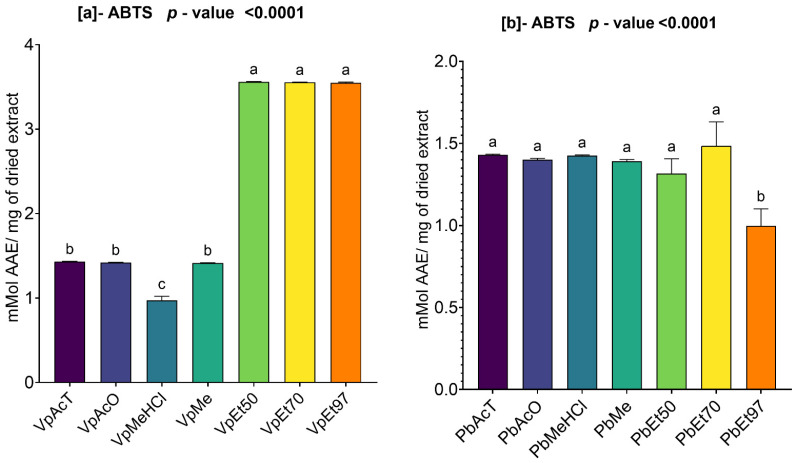
ABTS scavenging activity of *V. paradoxa* (**a**) and *P. biglobosa* (**b**) stem bark extracts. Means sharing the same coefficients a, b, or c are not statistically different.

Figure 8 summarizes the ABTS scavenging activity of the extracts from *V. paradoxa* and *P. biglobosa*.

The figure shows that the extracts of both plants demonstrated ABTS scavenging capacity. For the bark of *V. paradoxa*, the highest activities (3.5593 ± 0.00424 mmol AAE/mg of dry extract) were recorded in the ethanolic extracts across all percentages, while the lowest ABTS scavenging activity (0.9726 ± 0.03952 mmol AAE/mg of dry extract) was observed in the extract based on methanol with 1% HCl (Figure 8a). The analysis of variance (ANOVA) performed at the 95% significance level demonstrated that there is no statistically significant difference between the scavenging capacities of the ethanolic extracts, regardless of the percentage. The same applies to the extracts based on ethyl acetate, acetone, and methanol. However, all other observed differences are statistically significant (*p* value < 0.0001) (Figure 8a).

Similarly, the bark extracts of *P. biglobosa* exhibited a strong ABTS scavenging capacity. Apart from the extract based on 97% ethanol, which recorded the lowest scavenging activity (0.99 ± 0.084 mmol AAE/mg of dry extract), all other extracts exhibited scavenging activities ranging between 1.3 and 1.49 mmol AAE/mg of dry extract (Figure 8b). However, the ANOVA analysis at the 95% significance level demonstrated that, except for the extract based on 97% ethanol, which exhibited a statistically and significantly lower scavenging activity compared to all other extracts (*p* value < 0.0001), there is no statistically significant difference between the scavenging activities of the other extracts (Figure 8b).

#### 2.3.2. DPPH Scavenging Activity of Ethanolic Extracts of *V. paradoxa* and *P. biglobosa*

**Figure 9:**
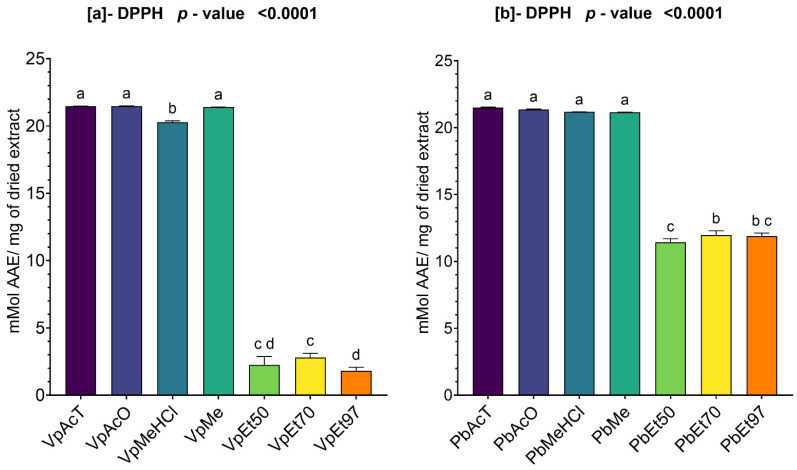
DPPH scavenging activity of ethanolic extracts from *V. paradoxa* (**a**) and *P. biglobosa* (**b**) stem bark. Means with the same letters a, b, c or d are not statistically different.

The DPPH scavenging activity of the bark extracts from *V. paradoxa* and *P. biglobosa* is shown in Figure 9. The figure indicates that extracts from both plants exhibited a strong DPPH scavenging ability.

For the bark of *V. paradoxa*, the extracts using ethyl acetate, acetone, methanol, and methanol with 1% HCl showed DPPH scavenging capacities ranging from 20 mmol AAE/mg to 22 mmol AAE/mg of dry extract. The extract based on 97% ethanol exhibited a DPPH scavenging capacity of 1.8 ± 0.21 mmol AAE/mg of dry extract (Figure 9a). An ANOVA analysis at the 95% significance level indicated no statistically significant difference between the ethanolic extracts, regardless of percentage, and no difference between the extracts based on ethyl acetate, acetone, and methanol. However, the other observed differences were statistically significant (*p* value < 0.0001) (Figure 9a).

For the stem bark extracts of *P. biglobosa*, the DPPH scavenging activity ranges from 11.43 ± 0.208 mmol AAE/mg of dry extract, recorded using the extract based on 50% ethanol, to 21.5 ± 0.02 mMol AAE/mg of dry extract, recorded using the extract based on ethyl acetate. It should be noted that the DPPH scavenging activities of the other types of extracts are almost double those of the ethanol-based extracts, regardless of the percentage (Figure 9b). At the 95% significance level, no statistically significant difference was observed between the DPPH scavenging capacities of the ethanolic extracts, regardless of the percentage. The same applies to extracts based on ethyl acetate, acetone, methanol, and methanol with 1% HCl. However, all other observed differences are statistically significant (Figure 9b).

#### 2.3.3. Ferric Reducing Antioxidant Power (FRAP) of Ethanolic Extracts of *V. paradoxa* and *P. biglobosa*

**Figure 10:**
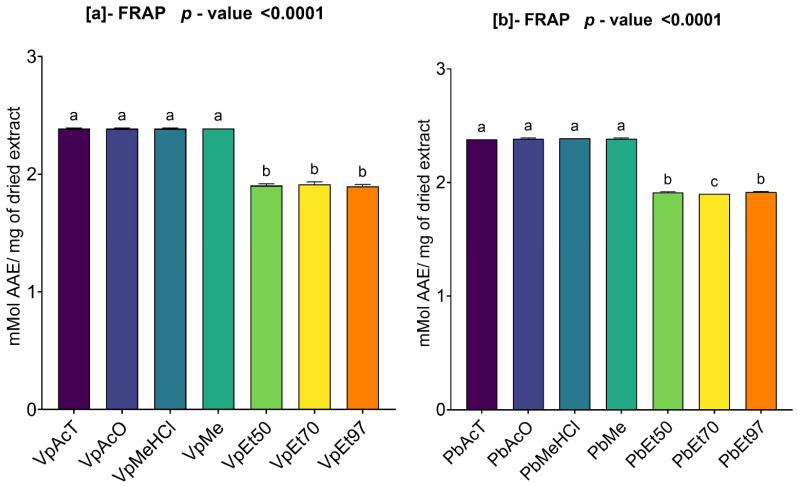
Ferric reducing antioxidant power (FRAP) activity of *V. paradoxa* (**a**) and *P. biglobosa* (**b**) stem bark extract. Means sharing the same letters a, b, or c are not statistically different.

Figure 10 summarizes the ferric ion reducing power (FRAP) results for the bark extracts of *V. paradoxa* and *P. biglobosa*.

The extracts of *V. paradoxa* showed a reducing power ranging from 1.89 ± 0.012 mmol AAE/mg of dry extract, recorded using the extract based on 97% ethanol, to 2.39 mmol AAE/mg of dry extract, recorded from the methanol-based extract. Using the ANOVA statistical test at the 95% confidence level, we observed that the ferric reducing powers of the extracts based on ethyl acetate, acetone, methanol, and methanol with 1% HCl, which are statistically similar, are significantly different from the ferric reducing powers of the ethanolic extracts, regardless of the percentage (*p* value < 0.0001). Furthermore, the ferric reducing powers of the ethanolic extracts, across all percentages, are statistically identical (Figure 10a).

Regarding the bark extracts of *P. biglobosa*, the highest reducing power is 2.39 mmol AAE/mg of dry extract, recorded by the extract based on methanol with 1% HCl, while the lowest reducing power, 1.9 mmol AAE/mg of dry extract, is observed in the extract based on 70% ethanol. Similarly, at the 95% significance level, the ANOVA test revealed that the ferric reducing powers of the extracts based on ethyl acetate, acetone, methanol, and methanol with 1% HCl, which are statistically similar, are significantly different from those of the ethanolic extracts, regardless of the percentage (*p* value < 0.0001). Also, the ferric reducing powers of the ethanolic extracts across all percentages are statistically similar (Figure 10b).

#### 2.3.4. Ammonium Phosphomolybdenum (APM) Activity Assay of Ethanolic Extracts from *V. paradoxa* and *P. biglobosa*

**Figure 11:**
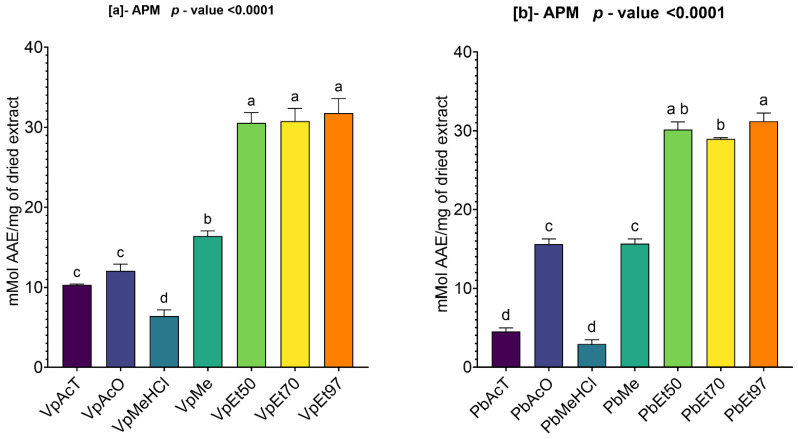
Phosphomolybdenum (APM) activity of ethanolic extract of *V. paradoxa* (**a**) and *P. biglobosa* (**b**) stem bark extract. Means with the same coefficients a, b, c, or d are not statistically different.

Figure 11 summarizes the ammonium phosphomolybdate (APM) reduction activity of *V. paradoxa* and *P. biglobosa bark extracts*. This figure shows that extracts from both plants, regardless of the solvent used, demonstrated a strong ability to reduce ammonium phosphomolybdate (APM).

On the one hand, the phosphomolybdate reduction activity of *V. paradoxa* bark extracts ranges from 6.41 ± 0.626 mmol AAE/mg of dry extract to 31.78 ± 1.481 mmol AAE/mg of dry extract. The highest activity (31.78 ± 1.481 mMol AAE/mg of dry extract) was recorded for the extract using 97% ethanol, while the lowest activity (6.41 ± 0.626 mmol AAE/mg of dry extract) was observed for the extract using methanol with 1% HCl (Figure 11a). The ANOVA test at a 95% significance level revealed no statistically significant difference between the activities of the ethanol extracts at all percentages and between the ethyl acetate and acetone extracts. However, the test indicated that the other observed differences are statistically significant (*p* value < 0.0001) (Figure 11a).

Conversely, the phosphomolybdate reduction activity of *P. biglobosa* bark extracts ranges from 2.94 ± 0.424 mmol AAE/mg of dry extract to 31.21 ± 0.852 mmol AAE/mg of dry extract. The highest activity was noted for the extract prepared with 97% ethanol, while the lowest was observed in the extract using methanol with 1% HCl (Figure 11b). At a 95% significance level, the ANOVA analysis found no statistically significant difference among the ethanol extracts at all percentages, between the ethyl acetate and methanol with 1% HCl extracts, and between the acetone and methanol extracts. However, all other differences observed are statistically significant (*p* value < 0.0001) (Figure 11b).

### 2.4. Protein Denaturation Inhibition (PDI) Activity Assay of V. paradoxa and P. biglobosa Extracts

**Figure 12:**
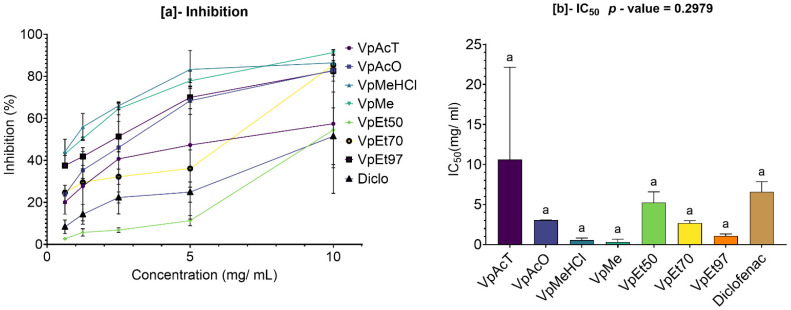
Protein denaturation inhibition activity of stem bark extract of *V. paradoxa* inhibition rate (**a**) and concentration for 50% inhibition (**b**). Means with the same coefficients a are not statistically different.

The results of evaluating the inhibition of protein denaturation by *V. paradoxa* are shown in Figure 12. This figure indicates that bark extracts and diclofenac exhibit a dose-dependent inhibitory effect on protein denaturation. This inhibitory effect was observed in all extracts, including diclofenac, starting at concentrations below 1 mg/mL. The methanol-based extracts and those using 1% HCl in methanol demonstrated the strongest inhibition, with higher inhibition rates than the others regardless of concentration. This is confirmed by their very low 50% inhibition concentrations, at 0.31 ± 0.244 mg/mL and 0.53 ± 0.193 mg/mL, respectively (Figure 12a). The lowest inhibition capacities were seen in the ethyl acetate and 50% ethanol extracts, which had lower inhibition rates than the others. This is reflected in their high 50% inhibition concentrations (IC_50_), at 5.21 ± 0.965 mg/mL and 10.61 ± 8.148 mg/mL, respectively. Compared to the extracts—except for those based on ethyl acetate and 50% ethanol—diclofenac showed lower activity (Figure 12b). However, at the 95% significance level, none of the observed differences are statistically significant (*p* value = 0.2979) (Figure 12b).

**Figure 13:**
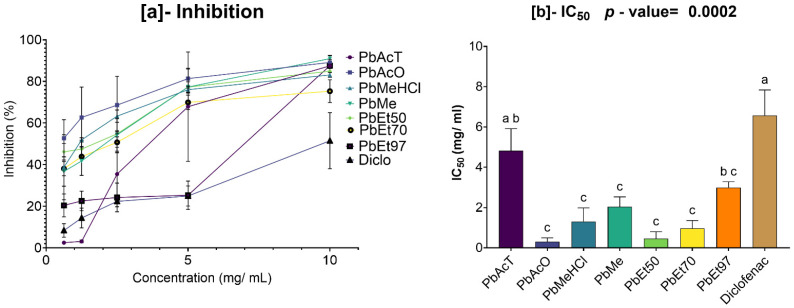
Protein denaturation inhibition activity of ethanolic extract of stem bark of *P. biglobosa* (**a**) concentration for 50% inhibition (**b**). Means with the same coefficients a, b, or c are not statistically different.

The results of the evaluation of the inhibition of protein denaturation by the bark extracts of *P. biglobosa* are presented in Figure 13. This figure shows that the bark extracts and diclofenac exerted a dose-dependent inhibitory effect on protein denaturation. This inhibitory activity was observed in all extracts, including diclofenac, starting at a concentration of about 0.5 mg/mL. The extracts based on acetone and 50% and 70% ethanol, followed by those based on methanol and 1% HCl in methanol, demonstrated the strongest inhibition capacities, with higher inhibition rates than the others, regardless of concentration. This is reflected in their very low 50% denaturation inhibition concentrations, 0.3 ± 0.134 mg/mL; 0.45 ± 0.24 mg/mL, and 0.96 ± 0.274 mg/mL for the acetone, 50% ethanol, and 70% ethanol extracts, respectively; and 1.3 ± 0.477 mg/mL and 2.04 ± 0.343 mg/mL for the methanol and 1% HCl in methanol extracts, respectively. The lowest inhibition capacities were observed in the extracts based on ethyl acetate and 97% ethanol, with lower inhibition rates than the others, as shown in (Figure 13a). This is also reflected in their very high 50% inhibition concentrations (IC_50_), at 4.82 ± 0.771 mg/mL and 2.99 ± 0.198 mg/mL. All the extracts showed a lower IC_50_ than that of diclofenac. However, at the 95% significance level, the differences in protein denaturation inhibition activity between diclofenac and the ethanolic extracts (regardless of percentage), the extract based on methanol, the extract based on 1% HCl in methanol, and the extract based on acetone are statistically significant (*p* value = 0.0002). The same applies to the extract based on ethyl acetate when compared to the other extracts, except for the methanol-based extract and the 97% ethanol-based extract. Nonetheless, the other observed differences are not statistically significant (Figure 13b).

### 2.5. Characterization of the Correlation Between the Different Pharmacological Properties and the Phenolic Compound Content of the Different Extracts of Each Plant Material

**Figure 14:**
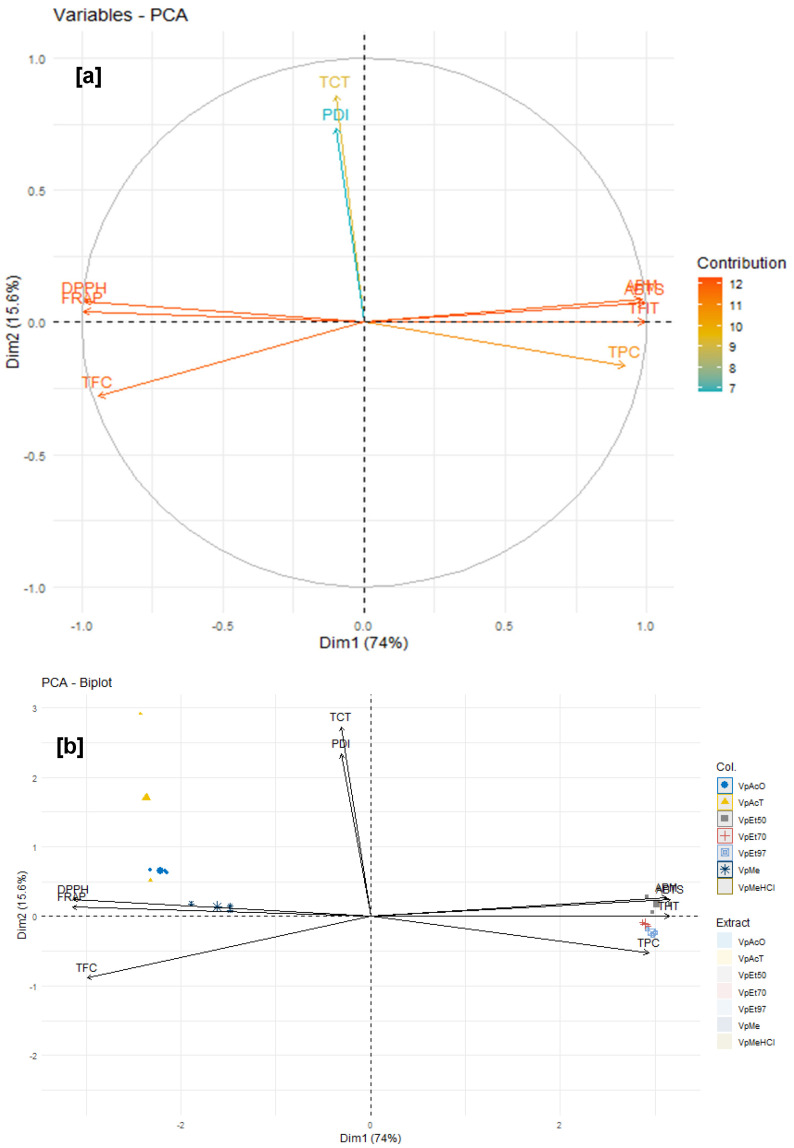
Correlation of pharmacological properties with phenolic content of *V. paradoxa* (**a**,**b**) stem bark extracts.

The data related to the phenolic compound content and the various activities tested were subjected to a principal component analysis, and the results are shown in Figure 14. The variables considered for this test are classified into two categories: the phenolic compound content and the pharmacological properties of the different extracts.

For the stem bark extracts of *V. paradoxa*, variables such as DPPH, FRAP, APM, and ABTS each contributed more than 12% to the explanation of the variances, followed by TFC, which demonstrated a contribution of 11%. Variables such as TPC and PDI each contributed approximately 10%, while TCT contributed 7% to the explanation of the total observed variances. Variables such as DPPH, FRAP, and TFC are strongly negatively correlated with the first dimension (Dim1), while APM, ABTS, THT, and TPC are positively and strongly correlated with this, contributing 76.3% to the explanation of the observed variances. The variables TCT and PDI, on the other hand, are respectively negatively and positively strongly correlated with the second dimension (Dim2), contributing 13.1% to the explanation of the variances. Thus, ferric reducing activity (FRAP), DPPH scavenging activity, and total flavonoid content, which are all strongly and positively correlated, are negatively and strongly correlated with phosphomolybdenum reduction activity, ABTS scavenging activity, total hydrolysable tannin content, and total phenolic content, which are, however, positively correlated with each other. Moreover, the condensed tannin content (TCT) and the protein denaturation inhibition activity (PDI) of *V. paradoxa* bark extracts, which have relatively weak correlations compared to the others, are negatively and strongly correlated (Figure 14a,b).

**Figure 15:**
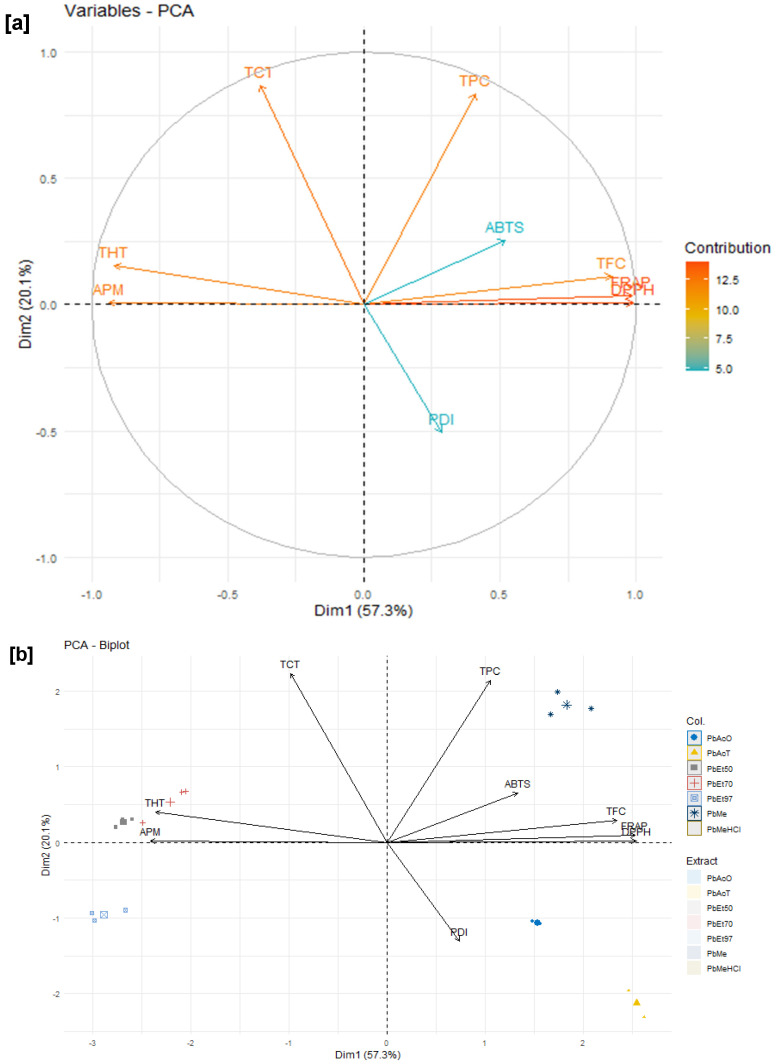
Correlation of pharmacological properties with phenolic content of *P. biglobosa* (**a**,**b**) stem bark extracts.

In addition, for the bark extracts of *P. biglobosa*, variables such as DPPH and FRAP each contributed more than 14% to the explanation of the variances, followed by TFC, THT, and APM, which demonstrated a contribution of approximately 12%. Variables such as PDI and TPC each contributed approximately 10% to the explanation of the total observed variances, while ABTS and TCT each contributed around 6%. Variables such as TCT, APM, and THT are strongly negatively correlated with the first dimension (Dim1), while ABTS, TFC, DPPH, and FRAP are positively and strongly correlated with it, contributing 57% to the explanation of the observed variances. The variables PDI and TPC are strongly negatively and positively correlated, respectively, with the second dimension (Dim2), contributing 19% to the explanation of the total variances. Thus, ferric reducing activity (FRAP), DPPH scavenging activity, ABTS scavenging activity, protein denaturation inhibition activity, total phenolic content, and total flavonoid content (TFC), which are all strongly and positively correlated, are negatively and strongly correlated with phosphomolybdenum reduction activity (APM), condensed tannin content (TCT), and hydrolysable tannin content (THT), which are, however, positively correlated with each other (Figure 15a,b).

### 2.6. Cytotoxicity of V. paradoxa and P. biglobosa Bark Extracts

**Figure 16:**
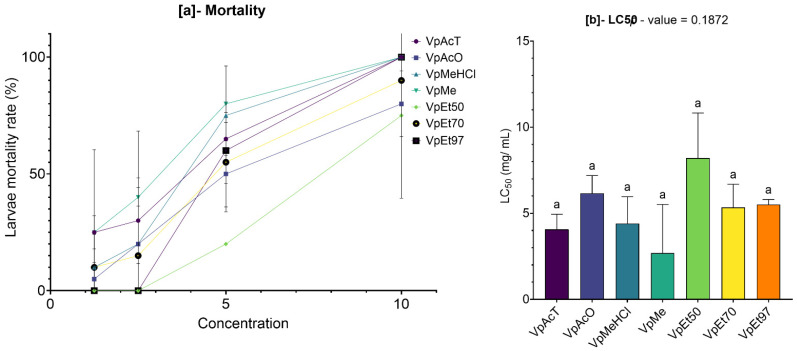
Cytotoxicity of *V. paradoxa* stem bark extracts on *Artemia salina* larvae: mortality (**a**); 50% mortality concentration (**b**). Means with the same coefficients a are not statistically different.

**Figure 17:**
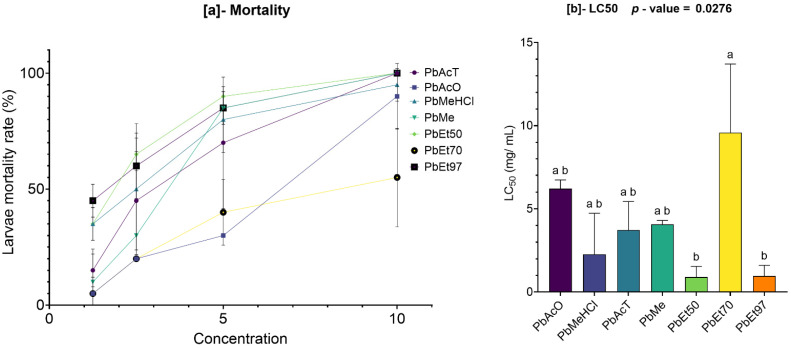
Cytotoxicity of *P. biglobosa* bark extracts on *Artemia salina* larvae: mortality (**a**); 50% mortality concentration (**b**). Means sharing the same coefficients a, b are not statistically different.

The cytotoxicity of the extracts was tested on *Artemia salina* larvae, and the results are displayed in Figure 16 and Figure 17.

On the one hand, the bark extracts of *V. paradoxa*, across all solvents, showed a dose-dependent mortality effect on the *Artemia salina* larvae (Figure 16). The mortality rates induced by the ethyl acetate and methanol-based extracts overlapped, ranging from 25 ± 5% and 25 ± 25% to 100 ± 0%, respectively. The same was true for the acetone-based extracts, methanol with 1% HCl, and 70% ethanol, whose mortality rates ranged from 5 ± 5%, 10 ± 10%, and 10 ± 10% to 80 ± 10%, 100 ± 0%, and 90 ± 0%, respectively. Furthermore, the methanol-based extracts and methanol with 1% HCl appeared to induce overlapping mortality rates. However, the 50% and 97% ethanol-based extracts only induced mortality starting at a concentration of 5 mg/mL, with mortality rates ranging from 20 ± 0% and 60 ± 10% to 75 ± 25% and 100 ± 0%, respectively. Nevertheless, the differences observed were not statistically significant (Figure 16a). The concentrations of each extract required to induce 50% mortality (LC_50_) in the exposed *Artemia salina* larvae were calculated, and the results revealed values ranging from 2.69 ± 1.99 mg/mL for the methanolic extract to 8.2 ± 1.84 mg/mL for the 50% ethanolic extract. These concentrations, both the minimum and maximum, are well above 0.1 mg/mL LC_50_ > 0.1 mg/mL according to the correlation grids linking the degree of toxicity to LC_50_ created by Moshi et al. [77], indicating an absence of cytotoxicity in the various extracts tested (Figure 16b).

Similarly, the bark extracts of *P. biglobosa*, across all solvents, showed a dose-dependent mortality effect on *Artemia salina* larvae (Figure 17). The 97% ethanolic extract caused the highest mortality, ranging from 45 ± 5% at 1.25 mg/mL to 100 ± 0% at 10 mg/mL. This was followed by tests using extracts based on methanol with 1% HCl and 50% ethanol, which, at the same concentration ranges, led to mortality rates from 35 ± 5% to 95 ± 5% and 100 ± 0%, respectively.

Although the 70% ethanolic extract appeared to obtain lower mortality rates, its effects were roughly similar to those caused by the acetone-based extracts, with values ranging from 5 ± 5% to 55 ± 15% and 90 ± 10%, respectively. A strong similarity was observed between the mortality caused by the ethyl acetate-based and methanol-based extracts, with values ranging from 10 ± 10% and 15 ± 5% to 100 ± 0% (Figure 17a). Except for the 50% and 97% ethanolic extracts, which recorded very low LC_50_ values (0.89 ± 0.45 mg/mL and 0.96 ± 0.45 mg/mL, respectively), the other extracts showed much higher LC_50_ values, with the highest being 9.57 ± 2.92 mg/mL for the 70% ethanolic extract. However, these variations were not statistically significant. These concentrations, both the minimum and maximum, are well above 0.1 mg/mL (LC_50_ > 0.1 mg/mL according to the correlation grids linking toxicity to LC_50_ by Moshi et al. (2004) [77]), indicating that the tested extracts are not cytotoxic (Figure 17b).

### 2.7. Acute Toxicity of V. paradoxa and P. biglobosa Extracts

#### 2.7.1. Body Modifications in Experimental Animals

**Figure 18:**
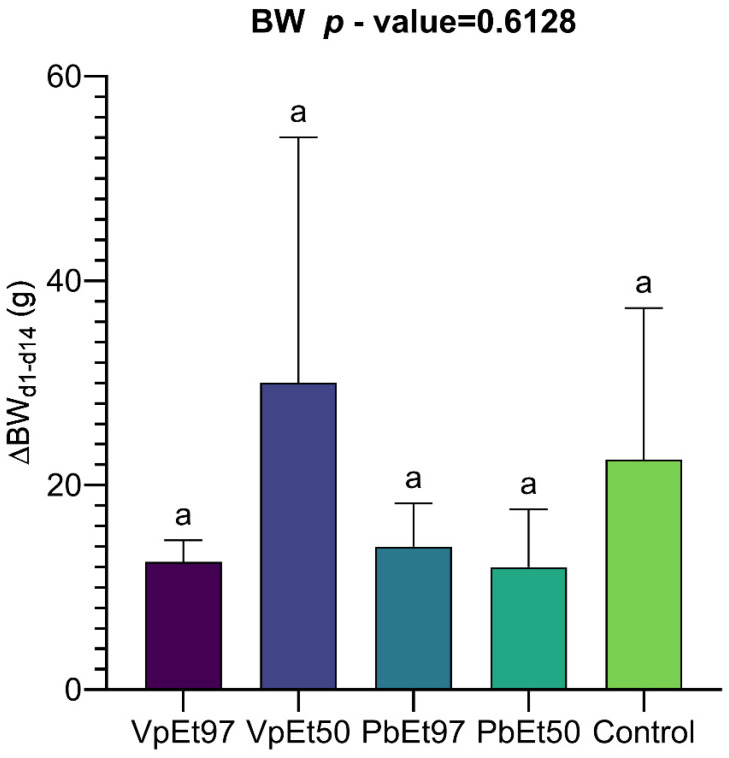
Changes in animal body weight during the 14-day follow-up of the acute toxicity test. Means sharing the same coefficients a are not statistically different.

Body weight changes were recorded every two weeks. No changes in skin, eyes, mucous membranes, or behavior were observed during the testing period. The average weight gains over the 14 days of the experiment are shown in Figure 18. This figure shows a weight gain ranging from 12 ± 4 g for the 50% ethanolic extract to 14 ± 3 g for the 97% ethanolic extract, both from *P. biglobosa*. The weight gain ranged from 12.5 ± 1.5 g for the 97% ethanolic extract to 30 ± 17 g for the 50% ethanolic extract, both from *V. paradoxa*. The placebo group experienced a weight gain of 22.5 ± 10.5 g. One-way analysis of variance indicated that the differences between the mean weights were not statistically significant.

#### 2.7.2. Hematological Monitoring

**Figure 19:**
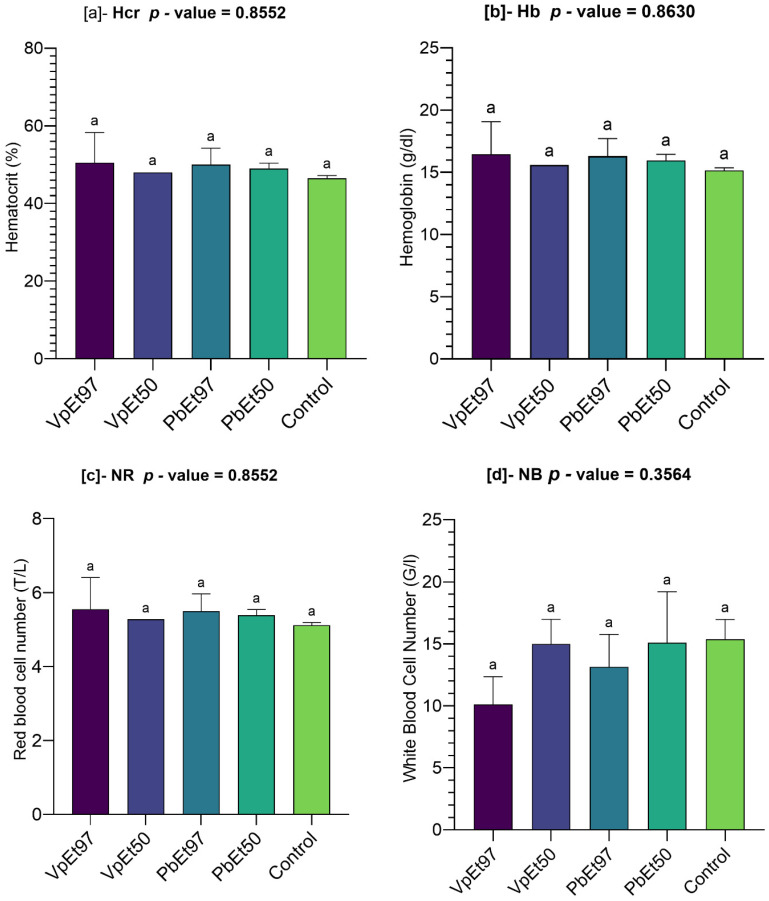
Hematological parameters of animals subjected to the acute toxicity test: hematocrit (**a**), hemoglobin (**b**), red blood cell count (**c**), and white blood cell count (**d**). Means marked with the same coefficients a are not statistically different.

The results of the hematological parameter measurements are shown in Figure 19. This figure illustrates the following:

The hematocrit level ranged from 48 ± 0% to 50.5 ± 5.5% for the 50% and 97% ethanolic extracts of *V. paradoxa* bark, respectively, while it ranged from 49 ± 1% to 50 ± 3% for the 50% and 97% ethanolic extracts of *P. biglobosa* bark, respectively. The control recorded 46.5 ± 0.5%, a slightly lower value than those recorded by the various extracts. However, analysis of variance indicated that these observed differences were not statistically significant (Figure 19a).

Minor variations were seen in the hemoglobin levels measured by the different extracts, including the control. Specifically, hemoglobin levels ranged from 15.6 ± 0 g/dl to 16.45 ± 1.85 g/dl for the 50% and 97% ethanolic extracts of *V. paradoxa* bark, respectively, while they ranged from 15.95 ± 0.35 g/dl to 16.3 ± 1 g/dl for the 50% and 97% ethanolic extracts of *P. biglobosa* bark, respectively. The control had a level of 15.15 ± 0.15 g/dl. These differences were not statistically significant (Figure 19b).

Similarly, the red blood cell counts ranged from 5.28 ± 0 T/L to 5.55 ± 0.6 T/L for the 50% and 97% ethanolic extracts of *V. paradoxa* bark, respectively, while they ranged from 5.39 ± 0.11 T/L to 5.5 ± 0.33 T/L for the 50% and 97% ethanolic extracts of *P. biglobosa* bark, respectively. The placebo group had a red blood cell count of 5.11 ± 0.05 T/L. Nonetheless, the differences between the mean red blood cell counts of the extracts and the control were not statistically significant (Figure 19c).

The white blood cell counts in the animals’ blood ranged from 10.11 ± 1.59 T/L to 15 ± 1.4 T/L for the 97% and 50% ethanolic extracts of *V. paradoxa* bark, respectively. For *P. biglobosa* bark extracts, the counts ranged from 13.15 ± 1.85 T/L to 13.15 ± 1.85 T/L for the 97% and 50% ethanolic extracts, respectively. The control group had a white blood cell count of 15.37 ± 1.13 T/L. One-way analysis of variance showed no significant differences between the mean white blood cell counts of the extracts and the control (Figure 19d).

**Figure 20:**
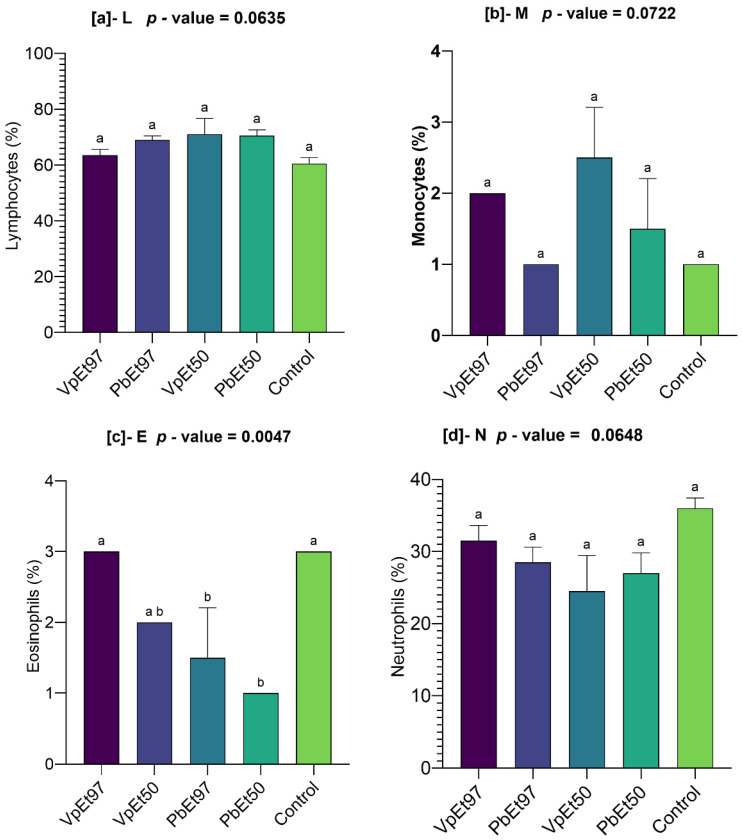
Immunological parameters of animals subjected to the acute toxicity test: lymphocytes (**a**), monocytes (**b**), eosinophils (**c**), and neutrophils (**d**). Means sharing the same letters a, b are not statistically different.

The levels of immune cells such as lymphocytes, monocytes, eosinophils, and neutrophils were measured, and the results are shown in Figure 20.

All extracts caused at least a 3% increase in lymphocyte levels compared to the control level, which was 60.5 ± 1.5%. The highest rate was observed with the 50% ethanolic extract of *V. paradoxa* bark (Figure 20a). In contrast to the 97% ethanolic extract of *P. biglobosa* bark, which had a monocyte rate similar to the control (1 ± 0%), the 50% and 97% ethanolic extracts of *V. paradoxa* bark and the 50% ethanolic extract of *P. biglobosa* bark showed monocyte rates of 2.5 ± 0.5%, 2 ± 0%, and 1.5 ± 0.5%, respectively (Figure 20b).

The 50% and 97% ethanolic extracts of *P. biglobosa* bark showed eosinophil rates that were significantly lower than the control. In comparison, the 50% and 97% ethanolic extracts of *V. paradoxa* bark recorded eosinophil rates of 3 ± 0% and 2 ± 0%, respectively. These rates, recorded for *V. paradoxa* bark extracts, were statistically similar to the control (Figure 20c).

With values of 31.5 ± 1.5%, 24.5 ± 3.5%, 28.5 ± 1.5%, and 27 ± 2%, the 97% and 50% ethanolic extracts of *V. paradoxa* bark, on the one hand, and *P. biglobosa* bark, on the other, recorded lower neutrophil rates than the control, which was 36 ± 1%. However, the ANOVA analysis showed no statistically significant differences between the observed mean rates (Figure 20d).

#### 2.7.3. Liver Function

**Figure 21:**
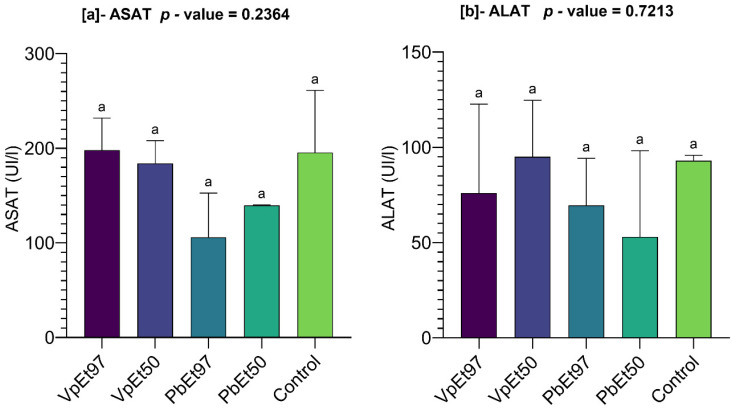
Liver function parameters of animals subjected to the acute toxicity test: AST (**a**); ALT (**b**). Means with the same letters a are not statistically different.

Liver function parameters like AST and ALT were measured, and the results are shown in Figure 21.

The AST levels ranged from 184 ± 17 U/L to 198 ± 24 U/L for the 50% and 97% ethanolic extracts of *V. paradoxa* bark, respectively, while they ranged from 106 ± 33 U/L to 139.5 ± 0.5 U/L for the 97% and 50% ethanolic extracts of *P. biglobosa* bark, respectively. Except for the 97% ethanolic extract of *V. paradoxa* bark, which recorded higher AST levels than the control (195.5 ± 46.5 U/L), the other extracts showed lower values. However, the differences observed between the mean AST levels were not statistically significant (Figure 21a).

Furthermore, ALT levels ranged from 76 ± 33 U/L to 95 ± 21 U/L for the 97% and 50% ethanolic extracts of *V. paradoxa* bark, respectively. In comparison, they ranged from 53 ± 32 U/L to 69.5 ± 17.5 U/L for the 50% and 97% ethanolic extracts of *P. biglobosa* bark, respectively. The 50% ethanolic extract of *V. paradoxa* bark recorded a higher ALT level than that of the control (93 ± 2 U/L), unlike the other extracts. Nevertheless, the differences observed between the mean ALT levels were not statistically significant (Figure 21b).

#### 2.7.4. Kidney Function

**Figure 22:**
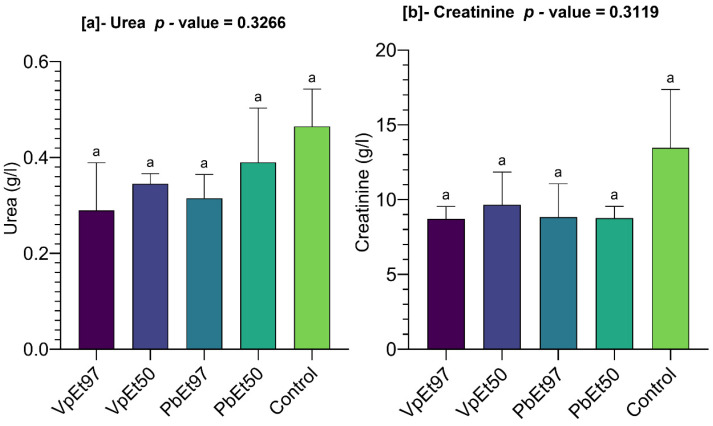
Kidney function parameters of animals subjected to the acute toxicity test: urea (**a**), creatinine (**b**). Means with the same coefficients a are not statistically different.

Kidney function was evaluated by measuring urea and creatinine levels, as illustrated in Figure 22.

The figure shows that blood urea levels ranged from 0.29 ± 0.07 g/L for the 97% ethanolic extract of *V. paradoxa* bark to 0.46 ± 0.05 g/L for the control. The urea levels recorded for all extracts were statistically lower than those of the control (Figure 22a). Similarly, creatinine levels ranged from 8.71 ± 0.58 g/L for the 97% ethanolic extract of *V. paradoxa* bark to 13.46 ± 2.76 g/L for the control. The creatinine levels for all extracts were lower than those of the control. However, these differences in both urea and creatinine levels were not statistically significant (Figure 22b).

#### 2.7.5. Relationships Between Hematological Parameters, Liver Function, Kidney Function, and the Different Extracts

**Figure 23:**
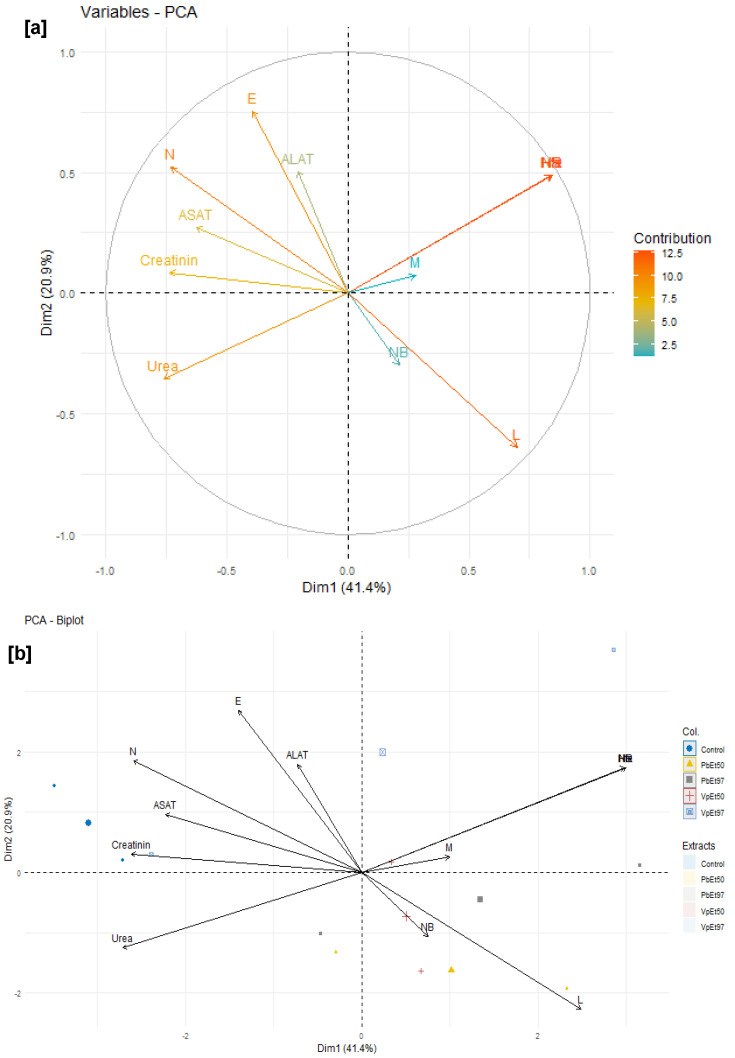
Correlation between hematological parameters, liver function, kidney function, and the different extracts: variables contribution (**a**), biplot (**b**).

Figure 23 characterizes the relationships between hematological parameters, the extracts, and the liver and kidney function of rats subjected to the acute toxicity test.

An examination of this figure reveals that the variables Hb (hemoglobin), Hcr (hematocrit), and NR (red blood cell count) contributed the most to the interpretation of the variances, with each accounting for more than 12.5%. They are followed by variables such as E (eosinophils), N (neutrophils), and L (lymphocytes), which each contributed approximately 10% (Figure 23a). NB (white blood cell count) and M (monocytes) recorded the lowest contributions, with no more than 2.5% each (Figure 23a).

The variables L, M, NR, Hcr, Hb, N, ASAT (aspartate aminotransferase), creatinine, and urea are strongly correlated with the first dimension, which accounts for 41.4% of the interpretation, compared to the second dimension, which accounts for only 20.9% (Figure 23a). Thus, the white blood cell count (NB), hematocrit level (Hcr), hemoglobin (Hb) level, red blood cell count (NR), lymphocyte (L) rate, and monocyte (M) rate, which increase more in subjects treated with the plant extracts, are strongly and positively correlated with each other (Figure 23b). The same applies to the levels of eosinophils, neutrophils, creatinine, urea, ASAT, and ALAT (alanine aminotransferase), which increase more in subjects treated with the placebo (Figure 23b).

### 2.8. Anti-Ulcer Activity of Stem Bark Extracts of V. paradoxa and P. biglobosa

#### 2.8.1. Stomach Protection Rate of Stem Bark Extracts of *V. paradoxa* and *P. biglobosa*

**Figure 24:**
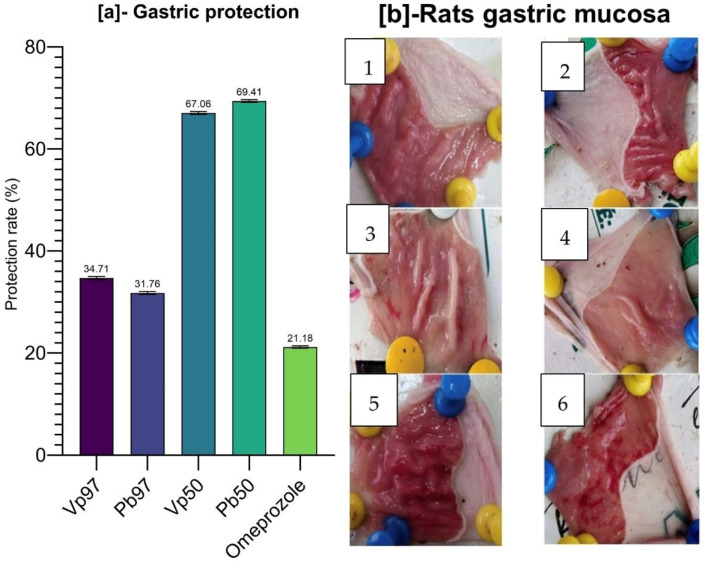
Degree of protection of rat stomachs against ulceration (**a**); sample macroscopical observation on gastric tissues of the experimental rat (**b**): b_1_: Vp97, b_2_: Pb97, b_3_: Vp50, b_4_: Pb50, b_5_: omeprazole, b_6_: distilled water.

The results of the protective activity of the extracts on the rats’ stomachs, expressed as a percentage, are shown in Figure 24a. From the figure, it is evident that the 50% ethanol extracts of the bark from both plants (*V. paradoxa* and *P. biglobosa*) exhibited the highest protection rates (67.06% and 69.41%, respectively) compared to the other extracts and the reference drug (omeprazole). The lowest protection rate was observed with omeprazole, the reference molecule, at 21.18%. The reported protection rate provides a good reflection of macroscopical observations of gastric tissues of the experimental rat (Figure 24b).

#### 2.8.2. Total Stomach Acidity of Gastric Ulcer Model Rats Treated with Stem Bark Extracts of *V. paradoxa* and *P. biglobosa*

**Figure 25:**
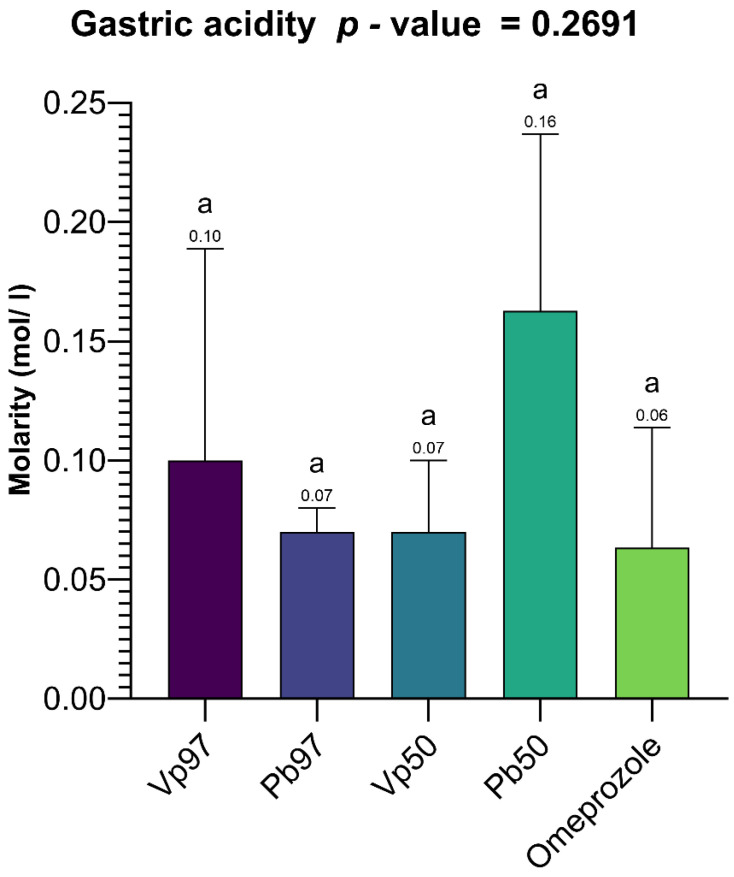
Total stomach acidity of gastric ulcer model rats treated with stem bark extracts of *V. paradoxa* and *P. biglobosa.* Means with the same coefficients a are not statistically different.

Figure 25 summarizes the results of the total gastric acidity measurements in rats. The data show that the highest total gastric acidity was recorded with the 50% ethanol extract of *P. biglobosa* bark (0.16 mol/L), followed by the 97% ethanol extract of *V. paradoxa* bark (0.1 mol/L). The lowest total gastric acidity was observed with omeprazole, the reference compound, at 0.06 mol/L. However, these variations in total gastric acidity across different treatments were not statistically significant. Specifically, the probability associated with the one-way ANOVA test, considering treatment as the factor influencing total gastric acidity, was *p* = 0.2691 (>0.05).

#### 2.8.3. Correlation Between Total Gastric Acidity and Protection Rate Against Gastric Ulcer

**Figure 26:**
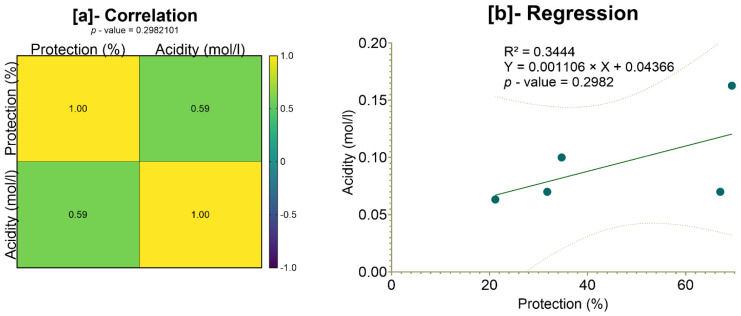
Correlation between total gastric acidity and protection rate against gastric ulcer: correlation (**a**), regression (**b**).

Figure 26 presents the results of the correlation test focusing on total gastric acidity and the stomach protection rate in rats. Although Pearson’s correlation analysis suggests a moderate correlation (r^2^ = 59%), the associated *p*-value (*p* = 0.2982101) exceeds the 5% threshold, indicating that this correlation is not statistically significant (Figure 26a). Similarly, the simple linear regression yielded an R^2^ of 34.44% with a slope of 0.001106, but again, the slope was not statistically significant (*p* = 0.2982). Therefore, we conclude that there is no statistically significant relationship between total gastric acidity and the protection rate (Figure 26b).

## 3. Discussion

Shea (*V. paradoxa*) and African locust bean (*P. biglobosa*) are two indigenous plants from semi-arid and arid regions, and they have been reported for their traditional uses: *V. paradoxa* [59,60], *P. biglobosa* [37,38,39,40,41]; their phytochemicals: *V. paradoxa* [39,45,46,56,58], *P. biglobosa* [42,43,44,78]; and their various biological activities: *V. paradoxa* [39,45,51,71,72,73,74,75,79], *P. biglobosa* [37,40,42,48,49,51,52,53,54]. This study screened the phytochemical content and determined the phenolic content and structures of the stem bark of these two plants. In addition, the in vitro antioxidant and anti-inflammatory activities of bark extracts were assessed, and the correlation between pharmacological activities and the phenolic content of the different extracts was analyzed.

A phytochemical screening of the bark powders of *V. paradoxa* and *P. biglobosa* revealed various groups of compounds in both plants. The bark of *V. paradoxa* and *P. biglobosa* contained phenolic compounds, including coumarins, tannins (gallic and catechin tannins), and flavonoids (anthocyanins and leuco-anthocyanins for *V. paradoxa* and anthocyanins for *P. biglobosa*). Other groups, such as saponins, terpenoid compounds (triterpenoids and mucilages), were detected. Alkaloids were found only in the bark powder of *V. paradoxa*, while glycosides (O-heterosides with reduced genin and C-heterosides) were found only in the bark powder of *P. biglobosa*. These results showed the rich phytochemical content of *V. paradoxa bark*. This aligns with findings by Abdullahi et al. [80], Manzoor et al. [11], and Namadina et al. [79], who detected classes of phytochemicals such as alkaloids, flavonoids, saponins, cardiac glycosides, tannins, steroids, triterpenes, phenols, and carbohydrates in *V. paradoxa* bark. Amlabu and Nock [70] did not detect flavonoids in *V. paradoxa* bark samples. *P. biglobosa* bark has been reported by Millogo-Kone et al. [81] and Abioye et al. [43] to contain major groups of secondary metabolites, including alkaloids, polyphenolics such as tannins, flavonoids, coumarins, anthocyanidins, saponins, steroids or sterols, glycosides, triterpenes, sugars, and reducing sugars.

Among these phytochemical groups, phenolics were quantified using spectrophotometric assays. The bark extracts from both plants showed higher levels of total phenolics, flavonoids, hydrolyzable tannins, and condensed tannins. Ethanol, at all concentrations, extracted more total phenolics from *V. paradoxa* bark than any other solvent, while methanol extracted more phenolics from *P. biglobosa* bark. Flavonoids were more effectively extracted with methanol containing 1% HCl in *V. paradoxa* bark, whereas they were better extracted with methanol, methanol with 1% HCl, and ethyl acetate in *P. biglobosa* bark. Condensed tannins were most effectively extracted with ethyl acetate, acetone, and methanol in *V. paradoxa* bark, while acetone and methanol were most effective in *P. biglobosa* bark. The most hydrolyzable tannins were extracted with all ethanol concentrations in *V. paradoxa* bark, and with 70% ethanol in *P. biglobosa* bark. Kagambega et al. [49] reported high polyphenol and flavonoid contents in methanolic and aqueous extracts of V. paradoxa bark. Similarly, Abdulazeez et al. [82] observed high levels of polyphenols, flavonoids, tannins, saponins, and alkaloids in the methanolic extract of *V. paradoxa* bark. Touré et al. [44] found elevated polyphenol and flavonoid levels in aqueous decoctions and macerations of *P. biglobosa* bark. However, these studies did not consider all extraction solvents, preventing them from identifying the optimal solvent for each phenolic group.

The bark extracts of both plants’ stem bark were analyzed, using HPLC-DAD for the accurate identification and measurement of phenolic compounds. The results revealed that *V. paradoxa* bark contained catechin, epicatechin, ferulic acid, apigenin-7-glucuronide, and hesperidin. Other researchers, like Foyet et al. [83], reported that *V. paradoxa* bark was rich in epicatechin, gallic acid, chlorogenic acid, and anthocyanins. Additionally, Kagambega et al. [49], using HPLC-UV-MS, detected gallic acid, protocatechuic acid, gentisic acid, catechin, vanillic acid, syringic acid, epicatechin, p-coumaric acid, and quercitrin. These authors identified compounds absent in our samples, whereas ferulic acid, present in our samples, was not reported by them. These differences likely result from variations in environmental conditions faced by the plants in their natural ecosystems, as they were collected from different geographical locations. The extract of *P. biglobosa* stem bark contained chlorogenic acid, catechin, caffeine, epicatechin, and cichoric acid. The catechins and epicatechins observed in *P. biglobosa* extracts support the findings of Tala et al. [42], who identified monomers and polymers of catechins using HPLC/ESI-IT-MS for proanthocyanidin analysis. Moreover, Kagambega et al. [49] reported gallic acid, protocatechuic acid, catechin, vanillic acid, syringic acid, epicatechin, p-coumaric acid, and ferulic acid in their HPLC-UV-MS analysis of P. biglobosa stem bark, detecting compounds not found in our extract. These phytochemical differences likely depend on environmental factors specific to the collection sites. The accumulation of secondary metabolites is heavily influenced by environmental variables such as light, temperature, soil moisture, fertility, and salinity. Variations in a single factor can alter the secondary metabolite profile of most plants [84,85]. The compounds identified may also depend on the extraction solvent; in this study, HPLC was performed only on the 50% methanol extract to maximize the extraction of polyphenolics. Recent studies emphasize that solvent polarity significantly affects the efficiency of phytochemical extraction. Since methanol is more polar than ethanol, it is generally more effective at extracting polar compounds like phenolics and flavonoids, yielding higher amounts of these compounds compared to ethanol and acetone [86,87]. To better understand the biological potential of *V. paradoxa* and *P. biglobosa* bark extracts, it is crucial to conduct HPLC-based screenings using various solvents. This approach will enable a more comprehensive identification and quantification of bioactive compounds, facilitating the development of more effective plant-based therapies. While methanol is commonly used to extract polar compounds such as phenolics and flavonoids, relying solely on methanolic extracts for HPLC analysis may not capture the full spectrum of bioactive compounds present in *V. paradoxa* and *P. biglobosa* stem bark. As shown by our results, the phenolic content of both species depends on the choice of extraction solvent, highlighting that different solvents, due to their varying polarities, can extract distinct groups of phytochemicals.

The antioxidant and anti-inflammatory properties were evaluated in vitro. Antioxidant activity was assessed using ABTS, DPPH, FRAP, and APM methods. All extracts showed strong antioxidant activity by scavenging ABTS and DPPH, and by reducing ammonium phosphomolybdenum (APM) and ferric ions (FRAP). The highest ABTS scavenging capacities were observed in ethanolic extracts of *V. paradoxa* bark, while *P. biglobosa* stem bark showed high activity across all extracts except those based on 97% ethanol. DPPH scavenging activity was notably strong in the bark of both plants across all extracts, except for the ethanol-based ones. Regarding APM reduction activity, the highest reduction was found in the bark of both plants compared to ethanol extracts. Conversely, a ferric reduction capacity (FRAP) was evident in the bark of both plants across all extracts, except those based on ethanol. The broad antioxidant spectrum and potent activity of *V. paradoxa* stem bark have been documented for aqueous and methanolic extracts, especially in terms of DPPH, ABTS, and FRAP scavenging properties [49,73]. This activity has been shown in vivo by improving scopolamine-induced memory impairments and reducing oxidative stress in the hippocampus of rats with methanolic extract [83]. Ethyl acetate, ethanolic, and aqueous extracts of *P. biglobosa* stem bark have been reported for their DPPH, hydroxyl radical scavenging, nitric oxide inhibition, and FRAP properties [53]. Additionally, aqueous decoctions and macerations of *P. biglobosa* bark have been noted for their ABTS scavenging ability [44].

Anti-inflammatory activity was tested in vitro using a protein denaturation inhibition assay. The methanol extract and methanol with 1% HCl showed the best inhibition of protein denaturation among all *V. paradoxa* bark extracts. However, for *P. biglobosa* bark, acetone and 50% ethanol extracts exhibited the highest denaturation inhibition activity. The methanolic extract of *V. paradoxa* bark has been previously reported for its anti-inflammatory and anti-arthritic effects in rat models at doses starting from 75 mg/kg body weight [64,67]. The strong anti-inflammatory activity of *V. paradoxa* and *P. biglobosa* extracts has also been documented by Kagambega et al. [49] in inflammation-induced rat models.

The reported link between antioxidant and anti-inflammatory activity and the phenolic content of bark extracts from both plants emphasizes the important role of flavonoids, tannins, and phenolic compounds in the antioxidant properties of *V. paradoxa* and *P. biglobosa* bark extracts. Phenolic compounds are generally recognized for their antioxidant and anti-inflammatory effects [88]. For instance, catechin, found in the bark of both plants, is known for these effects [89]. Additionally, the antioxidant and anti-inflammatory activities of hesperidin are well-documented, as reported by Parhiz et al. [90], Lorzadeh et al. [91], and Tejada et al. [92].

Phenolic compounds are not the only ones with antioxidant and anti-inflammatory activity; terpenoids are also studied, reported, and known for these activities [93,94,95,96,97,98,99,100,101,102]. Three modes of action have been identified so far. The main antioxidant mechanisms of terpenoids, such as carotenoids, include singlet oxygen-quenching, hydrogen transfer, and electron transfer [94]. Although alkaloids can act as both pro- and antioxidants, recent studies have reported a correlation between antioxidant activity and alkaloid content [103,104,105]. The anti-inflammatory effects of alkaloids have also been demonstrated and documented in recent research [106,107,108,109]. Additionally, glycosides can influence reduction and oxidation systems [110]. Our study detected the presence of terpenoids in the bark powders of *V. paradoxa* and *P. biglobosa*. Furthermore, alkaloids were found in the stem bark powder of *Vitellaria paradoxa*, while glycosides were identified in the stem bark powder of *Parkia biglobosa*. The presence of these metabolites is significant when assessing the antioxidant and anti-inflammatory activities of their extracts. To determine whether polyphenols are the sole contributors to these bioactivities, it is important to quantify these metabolite groups within the plant matrices and understand their specific roles in antioxidant and anti-inflammatory mechanisms. The health benefits of these antioxidants, along with their immunomodulatory effects, make them suitable for developing antioxidant-based therapeutics [111]. Yahfoufi et al. emphasized the importance of polyphenols as immunomodulators and anti-inflammatory agents [112]. The fact that *V. paradoxa* and *P. biglobosa* stem bark extracts showed higher polyphenol content and exhibited strong antioxidant and in vitro anti-inflammatory activities provides a trustworthy basis for further research.

The cytotoxic potential of plant extracts is a vital factor in assessing their safety profile, especially when they are meant for therapeutic purposes. In this study, the cytotoxicity of stem bark extracts of *V. paradoxa* and *P. biglobosa* was evaluated using the *Artemia salina* larvae lethality bioassay, a dependable initial method for predicting cytotoxicity.

Our results showed that both *V. paradoxa* and *P. biglobosa* stem bark extracts, regardless of the solvent used, had LC_50_ values significantly higher than the cytotoxicity threshold (LC_50_ > 0.1 mg/mL) proposed by Moshi et al. [77]. Although some extracts exhibited relatively low LC_50_ values, none fell below the critical threshold of 0.1 mg/mL, confirming their non-toxic nature in this model. In contrast to our findings, Ibrahim et al. [113] reported values well below this threshold and concluded that the methanolic extract of *Vitellaria paradoxa* leaf and stem bark showed moderate cytotoxicity. As noted in a previous review, the safety and toxicity of different parts of *V. paradoxa* remain unclear [39]. These variations could be due to differences in the geographical location of samples and environmental conditions. Conversely, Nounagnon et al. [114] reported LC50 values of 10.10 mg/mL and 19.90 mg/mL—slightly above the 0.1 mg/mL threshold—for ethanolic extracts of *Parkia biglobosa* stem bark and leaves, respectively, against Artemia salina larvae. The lack of cytotoxicity in both plant species’ extracts supports their safe use for further in vivo studies and potential therapeutic applications. While the *Artemia salina* assay offers valuable initial insights, it does not fully replicate mammalian toxicity; therefore, future toxicity evaluations using mammalian cell lines and long-term exposure models are essential to confirm these preliminary results.

After evaluating the cytotoxicity of the bark extracts using the *Artemia salina* model, which showed no significant toxic effects at the cellular level, an in vivo acute toxicity study was performed to further assess the systemic safety of the extracts. This phase involved monitoring physiological, hematological, immunological, hepatic, and renal parameters in treated animals to thoroughly evaluate potential toxic effects under physiological conditions, following OECD guidelines [115] for chemical safety assessments. Due to the potential toxicity linked to extracts prepared with solvents other than ethanol [116], we chose to assess the in vivo toxicity of ethanol-based stem bark extracts of *V. paradoxa* and *P. biglobosa* as a safer, representative alternative.

The evaluation of acute toxicity through weight-monitoring showed no significant adverse effects from the extracts of *V. paradoxa* and *P. biglobosa*. This supports the results reported by Mainasara et al. [117] regarding the absence of adverse effects on the weight gain of *V. paradoxa* stem bark extracts. The lack of significant differences in weight gain between treated and control groups suggests there is no acute systemic toxicity [118]. Additionally, the absence of changes in physical characteristics or behavior further supports the biocompatibility of the extracts at the doses used. Mainasara et al. [117] also reported no deaths or signs of toxicity in the oral acute toxicity test of *V. paradoxa* stem bark extract. Hematological parameters, including hematocrit, hemoglobin, and red and white blood cell counts, showed no significant differences between the control and treated groups. This aligns with the findings of Ayankunle et al., who observed that, except for platelet count, the administration of ethanolic V. paradoxa stem bark extract did not alter hematological parameters in the experimental model [119]. These findings suggest that the ethanolic extracts of both plants do not impair the hematopoietic system. The slight increases in lymphocyte levels observed with certain extracts may indicate mild immunostimulatory effects, although the lack of statistical significance tempers this interpretation. Nevertheless, plant secondary metabolites are well-known for their immunomodulatory effects [120,121], and further studies are needed to confirm this activity. Monocyte and eosinophil levels remained comparable to the control. Neutrophils, eosinophils, basophils, monocytes, and lymphocytes are white blood cells that play a crucial role in defending against infection and protecting the body from external threats [122]. This further supports the hematological safety of *P. biglobosa* and *V. paradoxa* stem bark extracts. Liver function markers (AST and ALT) remained within normal ranges, with no statistically significant differences across groups. Although there was a slight elevation in AST in animals treated with the 97% ethanolic *V. paradoxa extract*, the levels were comparable to the control. Overall, the data suggest that the extracts do not cause hepatotoxicity under the conditions of this study. This contradicts Ayankunle et al. [119], who reported that serum levels of creatinine, urea, ALAT, ASAT, and ALP were significantly higher in treated rats compared to controls when administered with ethanol extract of *V. paradoxa*. The non-toxic effects of *P. biglobosa* observed in this study are supported by the findings of Josiah et al. [123], who reported in a toxicity study of aqueous fractions of methanolic *P. biglobosa* stem bark extract in goats that there were no significant effects on biochemical parameters or changes in glucose, cholesterol, and triglyceride levels. Additionally, Udobi and Onaolapo [124] reported that the LD_50_ of *P. biglobosa* leaf and root extracts falls within 500–5000 mg/kg body weight, indicating slight toxicity but overall suggesting they are potentially safe [124]. However, seed extracts of *P. biglobosa* have been reported to cause a significant increase in hematological parameters such as white blood cells, lymphocytes, and neutrophils, as well as total protein, albumin, and aspartate aminotransferase, and a significant decrease in blood urea nitrogen and creatinine levels [125].

An assessment of kidney function showed no significant changes in urea and creatinine levels, which remained below those of the control group. This indicates preserved renal function and suggests that the tested doses of bark extracts did not harm kidney physiology. These findings are consistent with those of Mainasara et al. [126] and Ajibade and Soetan [125], who reported no significant differences between control and test groups except for urea and creatinine levels and indicated no harmful effects on kidney function in Wistar rats administered *P. biglobosa* leaf extracts and *V. paradoxa* stem bark extracts. Principal component analysis identified hemoglobin, hematocrit, and red blood cell count as the most influential parameters. These positively correlated parameters, which increase with plant extract administration, support the safety of the extracts. Conversely, higher levels of neutrophils, eosinophils, and liver/kidney function markers in the placebo group suggest no toxic effects from the extracts. Overall, these results support the non-toxic nature of *V. paradoxa* and *P. biglobosa* ethanolic bark extracts at the tested doses. This safety profile, combined with their previously shown biological activities, enhances the case for their potential use in therapy pending further sub-chronic and chronic toxicity studies. Our findings align with the study by Mainasara et al. [117] on the acute hepatotoxicity of methanol extract of *V. paradoxa* stem bark at doses of 5, 50, 300, 2000, and 5000 mg/kg, where no behavioral changes or mortality were observed in rats within 24 h and up to 14 days after treatment. Similarly, Ajibade and Soetan [125] reported that *P. biglobosa* leaf methanolic extract has no harmful effect on rat liver.

The low cytotoxicity observed in the *Artemia salina* model and the unremarkable acute toxicity reported for *V. paradoxa* indicate that the plant appears to be safe, as it has been shown to inhibit sodium arsenic toxicity in rats and Harwich fruit flies and to exhibit antiproliferative activity in cancer cell models [127]. A similar observation was made with *P. biglobosa* stem bark extract, which demonstrated hepatoprotective effects in preventing paracetamol-induced liver damage in Wistar rats [55].

*Vitellaria paradoxa* and *Parkia biglobosa* stem bark extracts showed significant polyphenolic content, which depended on the solvent’s nature and polarity. This content was strongly correlated with antioxidant activity and protein denaturation inhibition, likely due to their chemical structures. Given the absence of significant cytotoxic and acute toxic effects, we next evaluated the therapeutic potential of the ethanol-based extracts by testing their anti-ulcer effects in vivo. The stem bark extracts of *Vitellaria paradoxa* and *Parkia biglobosa* demonstrated promising gastroprotective properties in the ethanol-induced ulcer model. Notably, the 50% ethanolic extracts of both plants showed the highest stomach protection rates—67.06% for *V. paradoxa* and 69.41% for *P. biglobosa*—surpassing even the reference drug, omeprazole, which had a protection rate of only 21.18%. These results indicate a strong therapeutic potential for these extracts against gastric mucosal injury. Both *V. paradoxa* and *P. biglobosa* have been reported to exhibit remarkable biological activities, including anti-inflammatory, anticancer, and antioxidant effects, both in vitro and in vivo [38,39].

The observed gastroprotective effect may be linked to the presence of bioactive compounds such as flavonoids, tannins, and saponins, which are known for their antioxidant and cytoprotective properties [128,129,130]. Flavonoids, in particular, are recognized as strengthening the gastric mucosal barrier, inhibiting acid secretion, and scavenging free radicals, thus helping to reduce ulcer formation [129,130,131]. Tannins can precipitate proteins on the gastric lining, creating a protective layer that resists further irritation [129,130].

Although a moderate correlation (59%) was observed between total gastric acidity and the protection rate, the lack of statistical significance (*p* = 0.2982 > 0.05) suggests that acidity alone does not fully explain the extracts’ protective effects. This indicates the involvement of multiple mechanisms, potentially including enhanced gastric mucus secretion, antioxidant activity, anti-inflammatory effects, Gastric H^+^, K ^+^ ATPase inhibition, H_2_-antagonism, increased prostaglandin synthesis, gastric mucosa protection, and a combined or unknown mechanism of action [132], which warrants further investigation. Notably, the greater protection offered by the plant extracts compared to omeprazole highlights the potential of these natural compounds as effective alternatives or complementary agents in ulcer treatment. However, additional studies, including histopathological analyses, measurements of gastric mucus, and an assessment of inflammatory mediators, are needed to clarify the underlying mechanisms and establish their clinical relevance.

## 4. Materials and Methods

### 4.1. Plant Materials and Powder Preparation

The stem barks of *V. paradoxa* and *P. biglobosa* (Table 4) were collected from Northern Benin flora in Sèmèrè (Department of Donga) at 9°33′19.444″ N, 1°22′5.992″ W, dried under laboratory conditions, and powdered at the Laboratory of Biology and Molecular Typing in Microbiology at the University of Abomey-Calavi (UAC) in Benin. The various analyses were conducted at the Facultatea de Biotehnologii of the University of Agronomic Sciences and Veterinary Medicine of Bucharest in Romania and at the Laboratory of Biology and Molecular Typing in Microbiology at the University of Abomey-Calavi (UAC) in Benin.

### 4.2. Extracts Preparation

The *V. paradoxa* and *P. biglobosa* bark powder (50 g) was extracted via maceration following the protocol described by Phrompittayarat et al. with slight modification [133]. Methanol, ethyl acetate, acetone, methanol + 1% HCl, and ethanol (50%, 70%, and 97%) were used as extraction solvents. The dried plant material was macerated in 500 mL of solvent for 3 days (72 h) under stirring at room temperature and filtered through filter paper (Whatman no. 1). The obtained filtrate was evaporated using a rotary evaporator and dried in an incubator. The residue collected was stored in a refrigerator for subsequent analysis.

Extracts were prepared at 10 mg/mL by dissolving the residue in distilled water for the protein denaturation inhibition test; then, extracts were diluted to 1 mg/mL for measuring total phenolics, total flavonoids, condensed tannins, hydrolysable tannins, and antioxidant activities.

### 4.3. Phytochemical Screening of V. paradoxa and P. biglobosa Stem Bark

The phytochemical screening for the identification of tannins, flavonoids, coumarins, terpenes, alkaloids, etc., in both plant bark samples was carried out based on differential precipitation and colorimetric analysis using the method of Shaikh and Patil [134].

### 4.4. Determination of Polyphenolic Content in Extracts of V. paradoxa and P. biglobosa Stem Bark

#### 4.4.1. Determination of the Total Phenolic Content in the Stem Bark of *V. paradoxa* and *P. biglobosa*

The total phenol content was determined by spectrophotometry using gallic acid as a standard, following the method described by the International Organization for Standardization (ISO) 14502-1 [135] and adapted by Vamanu et al., with notable modifications [136]. Briefly, an aliquot of the diluted sample extract (0.25 mL) was transferred in triplicate to separate tubes containing a 1/10 dilution of Folin–Ciocalteu’s reagent in water (1.25 mL). Subsequently, 1.00 mL of sodium carbonate solution (7.5% *w*/*v*) was added. The tubes were then left to stand at room temperature for 15 min, and the absorbance at 765 nm was measured against water using a Helios Gamma UV–Visible Spectrophotometer (Thermo Fisher Scientific, Waltham, MA, USA). The concentration (X) of polyphenols in the diluted samples was calculated from a standard curve of gallic acid ranging from 100 to 700 μg/mL (y = 0.0013x + 0.0714; Pearson’s R2 = 0.9943). The total phenol content was expressed as micrograms of gallic acid equivalents per milligram of dry extract (µg GAE/mg dry extract) using Formula (1):

TPC = X × Ve/me(1)

where X = extract solution concentration in total phenolic content (µg GAE/mL); Ve = volume of extract used; me = mass contained in the volume of extract used (mg).

#### 4.4.2. Determination of Total Flavonoid Content in the Stem Bark of *V. paradoxa* and *P. biglobosa*

The total flavonoid content of the various extracts was quantified using a spectrophotometric method following the previously described protocol, with reasonable adjustments [137]. It involved mixing 2 mL of a 10-fold diluted extract solution (1 mg/mL) with 2 mL of a 2% AlCl_3_ solution. The absorbance was measured at 415 nm. A calibration curve (y = 0.0025x + 0.0465; R^2^ = 0.9928) was created using different concentrations (0.078 µg to 40 µg/mL) of quercetin as a standard, following the same procedure as the samples. All samples were analyzed in triplicate, and the results were expressed as micrograms of quercetin equivalent per milligram of dried extract (µg QE/mg dried extract) using Formula (2):

TFC = X × Ve/me(2)

where X = extract solution concentration in total flavonoids content (µg QE/mL); Ve = volume of extract used; me = mass contained in the volume of extract used (mg).

#### 4.4.3. Determination of Total Condensed Tannins Content in *V. paradoxa* and *P. biglobosa* Stem Bark Extracts

To determine the condensed tannin content, we used the method described by Leila et al. [138]. Briefly, 1 mL of sample, a 10-times diluted extract (1 mg/mL), was mixed with 2 mL of 1% vanillin (comprising 1 g of vanillin and 100 mL of 70% sulfuric acid) and incubated for 15 min in a water bath at 20 °C. The absorbance of the mixture was measured at 500 nm using a BioMATE 3S UV–visible spectrophotometer (Thermo Fisher Scientific, Waltham, MA, USA). The condensed tannin content of the samples was determined in triplicate, and the results were expressed as micrograms of catechin equivalents (µg CE) per mg of extract, calculated using a catechin calibration curve (y = 0.0003x + 0.0062; R^2^ = 0.99) plotted with catechin concentrations ranging from 100 to 1000 μg/mL.

The total condensed tannin content (TCT) was calculated using Formula (3):

TCT = X × Ve/me(3)

where X = extract solution concentration in total condensed tannins content (µg CE/mL); Ve = volume of extract used; me = mass contained in the volume of extract used (mg).

#### 4.4.4. Determination of Total Hydrolyzable Tannins Content in *V. paradoxa* and *P. biglobosa* Stem Bark Extracts

Total hydrolysable tannins were measured using the method of Mole and Waterman [139]. A total of 1 mL of a 10-fold diluted extract (1 mg/mL) was mixed with 3.5 mL of reagent (10^−2^ M ferric chloride FeCl_3_ in 10^−3^ M hydrochloric acid HCl). The absorbance was measured with a BioMATE 3S UV-visible spectrophotometer (Thermo Fisher Scientific, Waltham, MA, USA), at 660 nm after 15 min of incubation. The hydrolysable tannin content of the samples was determined in triplicate, and the results are expressed as mg gallic acid equivalent (GAE) per g of dried extract. The total hydrolysable tannin content (THT) was calculated according to Formula (4):

THT = A × MW × Ve/ε mole × me(4)

where A = absorbance; MW = weight of gallic acid (170.12 g/mol); Ve = volume of extract; ε mole = 2169 (gallic acid equivalence constant); me = mass contained in the volume of extract used (mg).

#### 4.4.5. Polyphenolic Profiling by HPLC-DAD of *V. paradoxa* and *P. biglobosa* Stem Bark

Further phenolic content determination of *V. paradoxa* and *P. biglobosa* stem bark was performed using an HPLC-DAD system with a similar method to that used by Kim et al. with slight modifications [140]. The methodology was validated by assessing linearity, range, LOD, LOQ, accuracy, precision, and content based on the Guidelines for Validation of Pharmaceutical Drugs from the Ministry of Food and Drug Safety, following their protocol.

First, 1 g of plant stem bark was extracted with 10 mL of 50% MeOH at reflux at 80 °C for 1 h. The mixture was cooled, filtered through Whatman no. 5 filter paper, and centrifuged at 8000 rpm for 10 min. The filtrate was dried under liquid nitrogen. The resulting residue was redissolved in 5 mL of MeOH. To analyze the sample composition, a Hitachi Chromaster HPLC system (Hitachi High-Tech, Tokyo, Japan) was used, equipped with a 5160 pump, 5310 column oven, 5260 thermostat autosampler, and 5430 DAD detector. Separation was performed on a ZORBAX SB-C18 4.6 × 150 mm, 3.5 µm column (ZORBAX^®^ Agilent Technologies, Inc. Santa Clara, CA, USA) An adapted RP-HPLC method was developed. The mobile phase consisted of a mixture of acetonitrile and methanol in a 1:1 ratio with 1% formic acid (A) and water with 1% formic acid (B). The gradient elution was carried out at 1 mL/min as follows: 0 min: 10% A—90% B; 5 min: 30% A—70% B; 20 min: 40% A—60% B; 25 min: 42.5% A—57.5% B; 26 min: 10% A—90% B; 30 min: 10% A—90% B. Standard stock solutions were prepared by dissolving reference standards in methanol, with concentrations ranging from 11.7 µg/mL to 193 µg/mL. If necessary, some samples were diluted with methanol before HPLC injection. All samples and standards were filtered through a 0.2 µm PTFE filter, and 5 µL of each solution was injected into the HPLC system. Data acquisition was performed at wavelengths of 320, 285, 267, and 369 nm.

### 4.5. Evaluation of Antioxidant Activity of V. paradoxa and P. biglobosa Stem Bark Extract

#### 4.5.1. ABTS (2,2′-Azinobis-[3-ethylbenzothiazoline-6-sulfonic acid]) Inhibition of *V. paradoxa* and *P. biglobosa* Stem Bark Extract

The ABTS radical scavenging assay was conducted following the method of Vamanu and Nita with slight modifications [141]. ABTS radical cations were generated by reacting ABTS (7 mM) with potassium persulfate (2.45 mM) in a 1:0.5 ratio and incubating the mixture at room temperature in the dark for 12 h. The resulting solution was then diluted with phosphate-buffered saline (PBS; pH = 7.4) to achieve an absorbance of 0.700 to 1.000 at 734 nm using a Helios Gamma UV-Visible Spectrophotometer (Thermo Fisher Scientific, Waltham, MA, USA). A 0.5 mL volume of 10-fold diluted extracts was added to 0.3 mL of the ABTS working solution and 0.2 mL of ethanol to reach a final volume of 1 mL. The absorbance was measured after 15 min in the dark at 734 nm with the Helios spectrophotometer. The percentage of inhibition was calculated using Equation (5):

% inhibition = [(Ac − As)/Ac] × 100(5)

where Ac = absorbance of control; As = absorbance of sample or standard molecule.

The scavenging activity (Sa) was calculated from the curve (y = 240.83x + 13.732; R^2^ = 0.9981) generated using ascorbic acid as a standard, and was then expressed as millimoles of ascorbic acid equivalent (mMol AAE) per mg of dried extract using Formula (6). The calibration curve was plotted with concentration on the *x*-axis and inhibition rate on the *y*-axis.

Sa = X × Ve/me(6)

where X = scavenging activity (mM AAE); Ve = volume of extract used; me = mass contained in the volume of extract used (mg).

#### 4.5.2. DPPH Scavenging Capacity of *V. paradoxa* and *P. biglobosa* Stem Bark Extracts

DPPH scavenging activity for several extracts was performed following the protocol used by Vamanu and Nita with slight modifications [141]. Briefly, 0.8 mL of 0.2 mM DPPH solution was mixed with 0.2 mL of the 10-fold diluted extract (1 mg) and 0.9 mL of absolute ethanol. The mixture was shaken and left to stand for 30 min in the dark. The absorbance was measured at 517 nm using a Helios Gamma UV–Visible Spectrophotometer (Thermo Fisher Scientific, Waltham, MA, USA). The DPPH radical scavenging activity (%) was calculated using Equation (7):

I% = 1 − (*As*/*Ac*) × 100 (7)

where *As* is the absorbance in the presence of sample and *Ac* is the absorbance in the absence of sample.

A standard curve (y = 46.998x − 1.9731; R^2^ = 0.9986) was created using a range of ascorbic acid concentrations, with concentrations on *the x*-axis and inhibition rate on the *y*-axis. The scavenging activity (Sa) was expressed as millimoles of Ascorbic Acid Equivalent (mMol AAE) per mg of dried extract using Formula (8):

Sa = X × Ve/me(8)

where X = scavenging activity (mM AAE); Ve = volume of extract used; me = mass contained in the volume of extract used (mg).

#### 4.5.3. Ferric-Reducing Antioxidant Power (FRAP) Activity of *V. paradoxa* and *P. biglobosa* Stem Bark Extracts

The Ferric-Reducing Antioxidant Power of various extracts was assessed using the protocol outlined by Vamanu and Nita [141]. Specifically, 2.5 mL of each 10-fold diluted extract (1 mg/mL) was combined with 2.5 mL of 200 mM sodium phosphate buffer (pH 6.6) and 2.5 mL of 1% potassium ferricyanide. This mixture was homogenized and incubated at 50 °C for 20 min. Next, 2.5 mL of 10% trichloroacetic acid was added, and the mixture was centrifuged at 3000 rpm for 10 min. The upper layer (2.5 mL) was then combined with 2.5 mL of deionized water and 0.5 mL of 0.1% ferric chloride. Absorbance was measured at 700 nm using a Helios Gamma UV–Visible Spectrophotometer (Thermo Fisher Scientific (formerly Thermo Electron Corporation), Waltham, MA, USA), which has a wavelength range of 190 to 1100 nm and a wavelength accuracy of 1 nm. A standard curve (y = 0.2447x + 38.435; R^2^ = 0.9903) was created using a range of concentrations of L-ascorbic acid. The reducing activity (Ra) was expressed as millimoles of Ascorbic Acid Equivalents (mMol AAE) per milligram of dried extract, using Formula (9).

Ra = X × Ve/me(9)

where X = reducing activity (mM AAE); Ve = volume of extract used; me = mass contained in the volume of extract used (mg).

#### 4.5.4. Ammonium Phosphomolybdenum (APM)-Reducing Activity of *V. paradoxa* and *P. biglobosa* Stem Bark Extracts

The phosphorus molybdenum assay was performed using the protocol outlined by Kedir et al. [142]. We prepared an aliquot of 0.1 mL of a 10-fold diluted extract (1 mg/mL) in triplicate assay tubes. Each aliquot was treated with 1 mL of reagent solution (0.6 M sulfuric acid, 28 mM sodium phosphate, and 4 mM ammonium molybdate). The tubes were incubated at 95°C in a water bath for 90 min. After cooling to room temperature, the absorbance was measured at 765 nm using a BioMATE 3S UV–visible spectrophotometer (Thermo Fisher Scientific, Waltham, MA, USA). Ascorbic acid served as the positive control to generate a standard curve (y = 1.4831x − 0.1568; R2 = 0.9957). The reducing activity was calculated from the standard curve and expressed as millimoles of Ascorbic Acid Equivalent (mMol AAE) per milligram of dried extract using Equation (10):

Ra = X × Ve/me(10)

where X = reducing activity (mM AAE); Ve = volume of extract used; me = mass contained in the volume of extract used (mg).

#### 4.5.5. Evaluation of the In Vitro Anti-Inflammatory Activity of *V. paradoxa* and *P. biglobosa* Stem Bark Extracts

In vitro anti-inflammatory activity was assessed using the heat-induced denaturation inhibition of egg albumin method of Chandra et al. [143]. The reaction involved mixing 0.2 mL of freshly laid egg albumin, 2.8 mL of phosphate-buffered saline (PBS, pH 6.4), and 2 mL of extract at various concentrations (0.625 to 5 mg/mL). A similar volume of distilled water served as the control. The mixture was incubated at (37 ± 2) °C in the oven for 15 min and then heated to 70 °C for 5 min. The absorbance was measured at 660 nm with a spectrophotometer after cooling to room temperature as measured using the water. Diclofenac sodium, at concentrations starting from half dilution (1.25 to 10 g/mL), was used as the reference molecule. The assay was performed in duplicate for each sample, and the percentage of denaturation inhibition was calculated using Formula (11):

% inhibition = 100 × (Vt/Vc − 1)(11)

where Vt = test absorbance of the sample; Vc = absorbance of the control.

The concentration of the extract or reference molecule for 50% inhibition (IC50) was determined by curve-fitting using the percentage inhibition as a function of concentration.

### 4.6. Evaluation of the Cytotoxicity of Extracts from V. paradoxa and P. biglobosa

The larval cytotoxicity of the extracts was evaluated using *Artemia salina* larvae according to the method applied by Chabi-Sika et al. [144].

The larvae were obtained by hatching 10 mg of *Artemia salina* eggs (Artemio^®^ JBL GmbH & Co.KG Dieselstraße, Neuhofen, Germany), kept under continuous agitation in 1 L of seawater for 72 h. A stock solution of 20 mg/mL of the extract was prepared (adding a small amount of DMSO if necessary for better dispersion or dissolution). From this solution, a series of successive dilutions was made in a ½ range of the extracts to be tested. A total of 1 mL of each geometric dilution was added to 1 mL of seawater containing 16 larvae. After 24 h of incubation, the dead, moribund, and live larvae were counted to determine the LC50. The LC50 was calculated from the regression line derived from the curve, showing mortality rate as a function of extract concentration. Each test was performed in duplicate, with a control consisting of 1 mL of seawater containing 16 larvae to which 1 mL of distilled water was added.

If there were deaths in the control group, the data were corrected using Abbott’s Formula (12):

% mortality = [(test − control)/control] × 100(12)

To interpret these results, correlation scales that connect the level of toxicity to the LC_50_ were proposed [77] (Table 5).

### 4.7. Evaluation of the Acute Toxicity of Extracts from V. paradoxa and P. biglobosa

#### 4.7.1. Animal and Ethical Approval

Female Wistar rats, specifically of the Exempt from Specific Pathogenic Organisms (EOPS) health status, were used in this study. These rats were about eight weeks old and weighed between 150 g and 200 g. This research protocol was approved by the Scientific Ethics Committee of the Doctoral School (Life Sciences) of the Faculty of Science and Technology (FAST) at the University of Abomey Calavi (UAC), Benin, under the number (UAC/FAST/EDSV/14168621).

#### 4.7.2. Experimental Design

The acute toxicity of the extracts was assessed in vivo using the 2000 mg/kg body weight limit dose test following the OECD method [115]. The selected rats were divided into five groups of four rats each (four rats per cage) and rats within each group were randomly assigned to different treatment groups. The control group received tap water (10 mL/kg body weight), while the other four groups received a single oral dose of plant extract at 2000 mg/kg body weight, as detailed in Table 6. Signs of toxicity were monitored at 1, 2, and 4 h after treatment, periodically during the first 24 h, and then daily for two weeks post-treatment. Changes in skin, eyes, mucous membranes, body weight, and behavior were recorded throughout the study period [115]. The treatments, data collection, and sampling were conducted in order of cage number.

#### 4.7.3. Monitoring of Body Changes

Body weight changes were tracked over a two-week period. Weight was measured on day 1 and again on day 14, which was the final day of the test. Changes in skin, eyes, mucous membranes, body weight, and behavior were observed throughout the test period [115].

#### 4.7.4. Hematological Monitoring

Hematological monitoring was performed using the method employed by Iserhienrhien and Okolie [145]. Specifically, the hematocrit level, hemoglobin content, white blood cell count, red blood cell count, lymphocyte, monocyte, eosinophil, and neutrophil counts were measured (BiopacBS-1100i, Shanghai, China).

#### 4.7.5. Liver Function

Following the strategy of Iserhienrhien and Okolie [145], dry tubes were used to collect blood samples, which were then centrifuged at 3000 rpm for 10 min at 5 °C to obtain serum isolates for liver condition analysis. The liver health of the animals after the experiment was assessed by measuring the activity of liver enzymes (ALAT, ASAT) using commercial kits, following the manufacturer’s protocol (Alpha Laboratories UK, London, UK).

#### 4.7.6. Kidney Function

Similarly, dry tubes were used to collect blood samples, which were then centrifuged at 3000 rpm for 10 min at 5 °C to obtain serum isolates for kidney function analysis. The condition of the animals’ kidneys after the experiment was assessed by measuring the concentrations of creatinine and urea using specific commercial kits, following the manufacturer’s protocol (Alpha Laboratories UK, London, UK) [145].

### 4.8. Evaluation of the Anti-Ulcer Activity of Stem Bark Extracts from V. paradoxa and P. biglobosa

The study used a model of ethanol-induced gastric ulcer in rats, following the method described by Shi et al. [146].

#### 4.8.1. Animals and Experimental Design

Eighteen male rats, approximately eight weeks old and weighing between 150 g and 200 g, were obtained from the Laboratory of Physiology and Experimental Pharmacology at the Faculty of Science and Technology, University of Abomey-Calavi. The procedures for animal care and use received approval from the Scientific Ethics Committee of the Doctoral School (Life Sciences) at the Faculty of Science and Technology (FAST), University of Abomey-Calavi (UAC), Benin. The approval, granted under the reference number UAC/FAST/EDSVT/14168621 on 15 January 2025, ensured compliance with ethical standards and regulations for animal research. All relevant institutional and governmental regulations concerning the ethical use of animals were followed. All animals were kept in a pathogen-free environment, fed ad libitum, and maintained at 24 ± 2 °C, with a relative humidity of 55 ± 5% and a 12-h light–dark cycle. A dose of 200 mg/kg body weight was used for the extract in the experiment.

**Figure 27:**
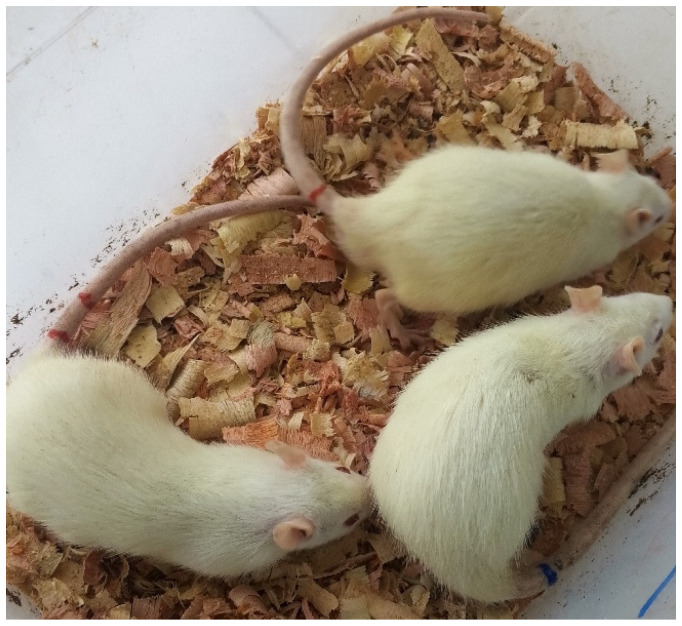
Animals’ acclimation.

The rats were allowed to acclimate for one week, after which they were divided into seven blocks (three rats per cage (Figure 27)), and then rats within each block were randomly assigned ck to treatment groups. They were fasted for 24 h before the experiment. Groups I, II, III, and IV received the extracts, as indicated in Table 7. Group V received omeprazole as a reference anti-ulcer drug at an oral dose of 20 mg/kg. Rats in groups VI and VII, representing the ulcer control and normal control groups, respectively, received distilled water.

Except for group VII, all groups received 5 mL/kg of absolute ethanol one hour after the oral administration of the extracts, distilled water, or omeprazole to induce a gastric ulcer.

One hour later, the animals were anesthetized with diethyl ether, sacrificed, and dissected to collect their stomachs. The gastric contents were separately collected, centrifuged at 5000 rpm for 10 min, and then stored in the refrigerator to measure the titratable acidity and mucin content. The stomachs were then washed with normal saline [147]. Treatments and sample collections were carried out according to the numerical order of the cages.

#### 4.8.2. Ulcer Index: Assessing Ulcer Size and Severity

The ulceration index was assessed using the method described by Ahmed et al. [148]. The stomachs were opened along the greater curvature and rinsed with normal saline to remove gastric contents and blood clots, then examined with a 10× magnifying glass to evaluate ulcer formation. The ulcer index can be determined using the scores outlined by Reddy et al. [149].

$$\begin{array}{l} \text{Normal-colored stomach --------- (0) Red coloration ------------------------------- (0.5) }\\ \text{Spot ulcer ----------------------------- (1) Hemorrhagic streak ---------------- (1.5) }\\ \text{Deep ulcers --------------------------- (2) Perforation ---------------------------- (3) } \end{array}$$

Ulcer Index (UI) = (UN + US + UP)/10(13)

where UI = ulcer index; UN = mean number of ulcers per animal; US = mean severity score; and UP = percentage of animals with an ulcer. The percentage of the protection ratio was calculated as follows (14):

% Protection = 100 − ([UI Treated]/[UI Control]) × 100(14)

#### 4.8.3. Determination of Total Gastric Acidity

A 1 mL sample of gastric juice was diluted with distilled water and placed in a 25 mL Erlenmeyer flask. Two drops of phenolphthalein indicator were added, and it was titrated with 0.01 N NaOH until a permanent pink color appeared. The amount of 0.01 N NaOH used was recorded. Total acidity was expressed in mEq/L and calculated using Formula (15):

Acidity = (V(NaOH) × N)/V_titrated(15)

where V_titrated = volume titrated; V(NaOH) = volume of NaOH added to reach equivalence; and N = normality.

### 4.9. Randomization and Blinding in Acute Toxicity and Gastroprotective Assay

Animals were randomly assigned to treatment groups through block randomization. The principal investigator decided on the allocation before the experiment began. Laboratory personnel, blinded to group assignments, administered treatments including extracts and collected samples to minimize performance bias. An independent researcher, also blinded to treatment groups, conducted outcome assessments such as clinical observations during the acute toxicity study and an evaluation of gastric lesions in the gastroprotective assay. Data analysis was carried out using anonymized datasets, with the analyst remaining unaware of group identities until the statistical analysis was completed.

### 4.10. Statistical Analysis

The data collected from our experiments were organized using Excel from the Microsoft Office 2016 package. GraphPad Prism^®^ software (version 9.3.0) was used for graph modeling and the statistical analysis of phenolic content, antioxidant and anti-inflammatory activities, toxicological monitoring parameters, and gastroprotective activity data. A one-way Analysis of Variance (ANOVA) was performed with Tukey or Dunnett post hoc tests to determine significant differences between the means. Principal Component and correlation analyses were also conducted using the FactoMineR and FactoExtra packages in R Studio 2025. All ANOVA and PCA tests were performed at the 95% significance level.

## 5. Conclusions

The bark of *V. paradoxa* and *P. biglobosa* were studied for their toxicity and various biological activities, including antioxidant, anti-inflammatory, and gastroprotective effects, based on different extraction solvents. The results showed that for both *V. paradoxa* and *P. biglobosa*, the nature and especially the polarity of the extraction solvent are key factors in efficiently targeting specific polyphenolic compounds. The HPLC-DAD analysis reported that *V. paradoxa* stem bark extracts contain catechin, epicatechin, ferulic acid, apigenin-7-glucuronide, and hesperidin, while *P. biglobosa* bark extract contains chlorogenic acid, catechin, caffeine, epicatechin, and cichoric acid. All extracts exerted strong antioxidant activity, which was found to depend on the phenolic groups contained within them. The extracts from both plants were found to be safe for *Artemia salina* and rat models, supporting their traditional use in treating various diseases. The extracts also demonstrated potent gastroprotective effects in rat models compared to omeprazole, a standard drug used in treating gastric ulcers. This underscores the importance of phytochemical diversity in the stem bark extracts of *V. paradoxa* and *P. biglobosa* and its influence on their antioxidant, anti-inflammatory, and gastroprotective activities. However, the choice of solvents can affect the variety of molecules extracted and, consequently, their biological activities. It would be valuable to analyze the phenolic profiles of other types of extracts, beyond methanolic ones, to better understand the molecular structures involved in these biological activities. Additionally, these extracts could be further explored for their immunomodulatory and antiproliferative effects on human gastrointestinal cancer cell lines and for in vivo evidence of their efficacy in inflammatory disease models.

## Figures and Tables

**Table 1:** Preliminary secondary metabolites profiling of the stem bark of *V. paradoxa* and *P. biglobosa*.

Classes of Secondary Metabolites		Secondary Metabolites	*V. paradoxa* Stem Bark	*P. biglobosa* Stem Bark
Polyphenols	Tannins	Gallic tannins	+	+
		Catechic Tannins	+	+
	Flavonoids	Flavonoids	+	+
		Anthocyanes	+	+
		Leuco-anthocyanes	+	−
	Coumarins		+	+
Quinonic compounds			−	−
Saponosides			+	+
Terpenic compounds		Triterpenoids	+	+
Cyanogenic compounds			−	−
Mucilage			+	+
Reducing compounds			−	−
Free anthracenic compounds			−	−
Glycosides	Heterosides	O-heterosides	−	−
		reduced genin O-heterosides	−	+
		C-heterosides	−	+
Alkaloids			+	−

+: means present; −: means absent.

**Table 2:** HPLC-DAD summary of stem bark of *V. paradoxa*.

Rt (min)	UV λmax (nm)	Area	Phenolic Compound	Content (µg/g of Plant Powder)
6.334	285	37.735	Catechin	0.0641
7.451	285	218.255	Epicatechin	0.3848
9.913	320	14.086	Ferulic acid	˂LOQ
12.457	267	41.563	Apigenin-7-gluc	0.012
12.458	285	41.282	Hesperidin	˂LOQ

LOQ: means limit of quantification; Rt: retention time.

**Table 3:** HPLC-DAD summary of the stem bark of *P. biglobosa*.

Rt (min)	UV λmax (nm)	Area	Phenolic Compound	Content (µg/g of Plant Powder)
6.033	320	20.074	Chlorog acid	0.0055
6.035	285	30.831	Catechin	0.0529
7.035	267	32.066	Caffeine	˂LOQ
7.198	285	21.707	Epicatechin	0.0423
10.756	320	47.899	Cichoric acid	0.0166

LOQ: means limit of quantification; Rt: retention time.

**Table 4:** Plants name.

Latin Name	French Name	English Name	Local Name in Benin
*V. paradoxa*	Karité	Shea	in Fongbé: Limi; in Adjagbé: YƆku
*P. biglobosa*	Néré	cowpea	in Fongbé: Hua; in Adjagbé: Ewa

**Table 5:** Relationship between LC_50_ and toxicity.

LC50	Toxicity
LC50 > 0.1 mg/mL	− (Non-toxic)
0.1 mg/mL > LC50 ≥ 0.050 mg/mL	+ (Low toxicity)
0.050 mg/mL > LC50 ≥ 0.01 mg/mL	++ (Moderate toxicity)
LC50 < 0.01 mg/mL	+++ (High toxicity)

**Table 6:** Design of the acute toxicity test for bark extracts of *V. paradoxa* and *P. biglobosa*.

Groups	Treatments
Group 1	2000 mg/kg body weight of VpEt97
Group 2	2000 mg/kg body weight of PbEt97
Group 3	2000 mg/kg body weight of VpEt50
Group 4	2000 mg/kg body weight of PbEt50
Group 5	10 mL/kg body weight of distilled water

**Table 7:** Design of the anti-ulcer test for bark extracts of *V. paradoxa* and *P. biglobosa*.

Groups	Treatments	Ulcer Induction
Group 1	200 mg/kg body weight of VpEt97	5 mL ethanol/kg body weight
Group 2	200 mg/kg body weight of PbEt97	5 mL ethanol/kg body weight
Group 3	200 mg/kg body weight of VpEt50	5 mL ethanol/kg body weight
Group 4	200 mg/kg body weight of PbEt50	5 mL ethanol/kg body weight
Group 5	20 mg/kg body weight of omeprazole	5 mL ethanol/kg body weight
Group 6	1 mL/100 mg body weight of distilled water	5 mL ethanol/kg body weight
Group 7	1 mL/100 mg body weight of distilled water	Not induced

## Data Availability

The raw data supporting the conclusions of this article will be made available by the authors on request.

## Abbreviations

The following abbreviations are used in this manuscript:

•OH	Hydroxyl radical
^1^O_2_	Singlet oxygen
AAE	Ascorbic acid equivalent
ABTS	2,2-azino-bis (3-ethylbenzothiazoline-6-sulfonic acid)
AcO	Acetone
AcT	Ethyle acetate
ALAT/ALT	Alanine aminotransferase
AlCl_3_	Aluminum chloride
ANOVA	Analysis f variance
APM	Ammonium phosphomolybdate
ASAT/ASP/AST	Aspartate amino-transferase,
CE	Catechin equivalent
CUPRAC	CUPric-reducing antioxidant capacity
DPPH	2,2-diphenyl-1-picrylhydrazyl.
Et	Ethanol
FeCl_3_	Ferric chloride
FRAP	Ferric-reducing antioxidant power
GAE	Gallic acid equivalent
H^+^	Hydrogen ion
H_2_O_2_	Hydrogen peroxide
Hb	Hemoglobin
HCl	Hydrochloric acid
Hcr	Hematocrit
HPLC/ESI-IT-MS	High-performance liquid chromatography with electrospray ionization and ion trap mass spectrometry
HPLC-DAD	High-performance liquid chromatography with diode array detection
HPLC-UV-MS	High-performance liquid chromatography with ultraviolet detection and mass spectrometry
IC_50_	Inhibitory concentration 50%
K^+^	Potassium ion
L	Lymphocyte
LC_50_	Lethal concentration 50%
LOD	Limit of detection
LOQ	Limit of quantification
M	Monocyte
Me	Methanol
MeHCl	Methanol with 1% of HCl
N	Neutrophil
NB	Nombre de globule blanc (white blood cell count)
NR	Nombre de globule rouge (red blood cell count)
NRF2	Nuclear Factor erythroid 2-Related factor 2
O^−2^	Superoxide
OECD	Organisation for Economic Co-operation and Development
PDI	Protein denaturation inhibition
QE	Quercetin equivalent
RNS	Reactive nitrogen species
ROS	Reactive oxygen species
RP-HPLC	Reverse-phase high-performance liquid chromatography
TCT	Total condensed tannin
TFC	Total flavonoid content
THT	Total hydrolysable tannin
TPC	Total phenolic content
U/L	Units per liter
